# Sphk2 deletion is involved in structural abnormalities and Th17 response but does not aggravate colon inflammation induced by sub-chronic stress

**DOI:** 10.1038/s41598-022-08011-8

**Published:** 2022-03-08

**Authors:** David Martín-Hernández, Irene L. Gutiérrez, Marta González-Prieto, Karina S. MacDowell, Javier Robledo-Montaña, Hiram Tendilla-Beltrán, Natalia Calleja-Rodríguez, Álvaro G. Bris, Cristina Ulecia-Morón, Beatriz Moreno, Javier R. Caso, Borja García-Bueno, Sandra Rodrigues-Mascarenhas, Ignacio Marín-Jiménez, Juan Carlos Leza, Luis Menchén

**Affiliations:** 1 Servicio de Psiquiatría del Niño y del Adolescente, Instituto de Psiquiatría y Salud Mental, Hospital General Universitario Gregorio Marañón, Facultad de Medicina, Universidad Complutense, Instituto de Investigación Sanitaria Gregorio Marañón, Doctor Esquerdo 46, 28007 Madrid, Spain; 2 CIBERSAM, Madrid, Spain; 3 Departamento de Farmacología y Toxicología, Facultad de Medicina, Universidad Complutense, Imas12, IUIN, Madrid, Spain; 4 Laboratorio de Neuropsiquiatría, Instituto de Fisiología, Benemérita Universidad Autónoma de Puebla (BUAP), Puebla, Mexico; 5 Laboratório de Imunobiotecnologia, Centro de Biotecnologia, Universidade Federal da Paraíba (UFPB), João Pessoa, Brazil; 6 Servicio de Aparato Digestivo, Hospital General Universitario Gregorio Marañón, Departamento de Medicina, Universidad Complutense, Instituto de Investigación Sanitaria Gregorio Marañón, Madrid, Spain; 7 CIBEREHD, Madrid, Spain

**Keywords:** Inflammatory bowel disease, Preclinical research, Molecular biology, Inflammation, Mucosal immunology

## Abstract

The chronic inflammatory process that characterizes inflammatory bowel diseases (IBD) is mainly driven by T-cell response to microbial and environmental antigens. Psychological stress is a potential trigger of clinical flares of IBD, and sphingosine-1-phosphate (S1P) is involved in T-cell recruitment. Hence, stress impact and the absence of sphingosine kinase 2 (Sphk2), an enzyme of S1P metabolism, were evaluated in the colon of mice after sub-chronic stress exposure. Here, we show that sub-chronic stress increased S1P in the mouse colon, possibly due to a decrease in its degradation enzymes and Sphk2. S1P accumulation could lead to inflammation and immune dysregulation reflected by upregulation of toll-like receptor 4 (TLR4) pathway, inhibition of anti-inflammatory mechanisms, cytokine-expression profile towards a T-helper lymphocyte 17 (Th17) polarization, plasmacytosis, decrease in IgA+ lymphoid lineage cells (CD45+)/B cells/plasmablasts, and increase in IgM+ B cells. Stress also enhanced intestinal permeability. Sphk2 knockout mice presented a cytokine-expression profile towards a boosted Th17 response, lower expression of claudin 3,4,7,8, and structural abnormalities in the colon. Intestinal pathophysiology should consider stress and S1P as modulators of the immune response. S1P-based drugs, including Sphk2 potentiation, represent a promising approach to treat IBD.

## Introduction

Evidence in inflammatory bowel diseases (IBD), such as ulcerative colitis (UC) and Crohn's disease (CD), has led to immune-based therapies to control symptomatology^1^. However, refractory patients remind us of the pressing need for a better understanding of immune implications in the IBD pathophysiology to unravel new pharmacological targets.

An immune dysregulation could cause oxidative/nitrosative damage to the intestinal epithelial cells, precipitating an intestinal barrier dysfunction or *leaky gut*^2^. As a result of altered immune response to environmental antigens, including microbes, chronic inflammation represents the main feature of IBD. Significantly, beyond the well-known and demonstrated stress effects on the immune system, most experimental evidence indicates that stress may also impact the clinical course of IBD^3^. Stress is related to IBD symptomatic activity^4,5^, but its impact on objective measures of intestinal inflammation such as fecal calprotectin is not clear^4^. However, the complex molecular network involved in stress-immune interplay in the colon is not fully deciphered.

Sphingosine-1-phosphate (S1P) emerges as a promising pathway to modulate intestinal inflammatory processes, as demonstrated by encouraging results of phase II clinical trials targeting S1P receptor subtypes 1 and 5 (S1PR1/5)^6^. S1P is a bioactive lipid that promotes immune cell recruitment and contributes to inflammation, among other cellular functions. Metabolism of S1P comprises two synthesis sphingosine kinases (Sphk1 and Sphk2) and both reversible and irreversible degradation systems. Sphingosine-1-phosphate phosphatases (SGPP1 and SGPP2) are responsible for the reversible conversion into sphingosine, while sphingosine-1-phosphate lyase 1 (SGPL1) irreversibly transforms S1P into hexadecenal and ethanolamine-phosphate. The biological activity of S1P is exerted through 5 types of G protein-coupled receptors. S1PR1, 2, and 3 are the most studied and ubiquitously expressed—also in the colon—, while S1PR4 seems to be more specific of hematopoietic/lymphatic tissues and S1PR5 of the central nervous system^7^. All the elements of the S1P pathway are under the scope of research not only in IBD but also in gastrointestinal cancers owing to the involvement of S1P in the tumor microenvironment^8,9^. In the same vein, psychological stress can modulate S1P signaling, and its receptors have been proposed as pharmacological targets due to their capacity to control neuroinflammation^10^.

Particularly, Sphk2 plays an intriguing part in S1P metabolism due to a controversial dual role described for Sphk2 inhibitors. The most accepted hypothesis defends that Sphk2 inhibition or gene deletion causes a counterintuitive increase in S1P blood levels^11^, but there are also studies showing a decrease in S1P^12^. The mechanism underlying this discrepancy remains still unknown, but a compensatory increase in Sphk1 activity or a lower ability to remove S1P could explain the upregulation of S1P after Sphk2 blocking^11,13^. Moreover, Sphk2 and its potential as a pharmacological target are currently under investigation for cancer, neurodegenerative, cardiovascular, and inflammatory diseases^14^.

Our experimental setting pursues two primary goals. Firstly, an exhaustive characterization of the stress-driven immunological effects—both innate and adaptative—in the colon, including the role of S1P pathways and their consequences for intestinal permeability after a sub-chronic stress protocol in mice. And secondly, we delve into the Sphk2 role using knockout mice to shed light on its contribution to the stress-immune response and intestinal barrier function.

## Results

### Sphk2 deletion did not modify the general stress-exposure effects of weight loss and corticosterone upregulation but decreased the spleen size

**Figure 1:**
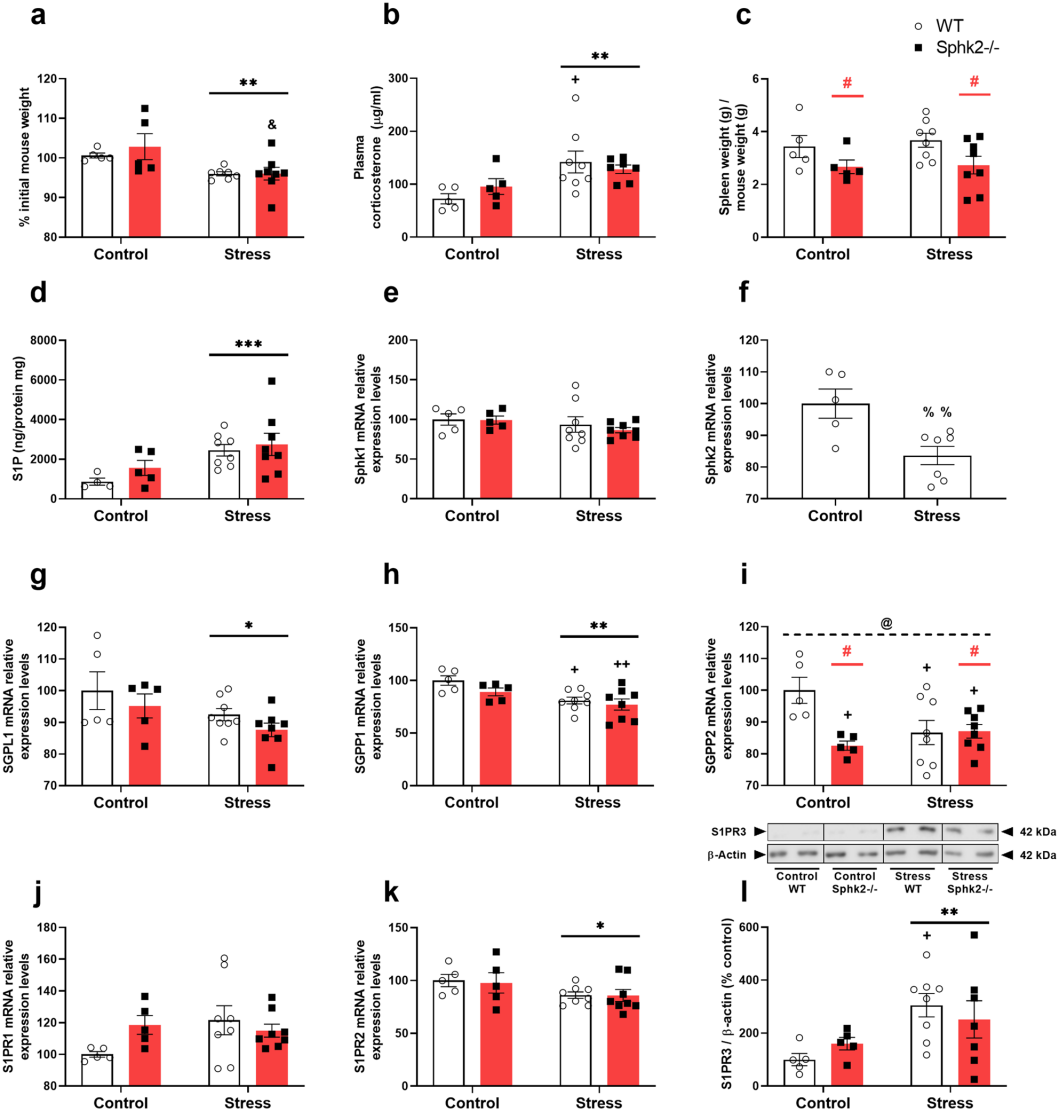
General stress effects and S1P pathways analyses in the colon of WT and Sphk2 mice after sub-chronic stress exposure. Percentage of weight gain after the stress protocol (**a**), plasma corticosterone levels (**b**), relative spleen weight (**c**), colon S1P levels (**d**), Sphk1 mRNA levels (**e**), Sphk2 mRNA levels (**f**), SGPL1 mRNA levels (**g**), SGPP1 mRNA levels (**h**), SGPP2 mRNA levels (**i**), S1PR1 mRNA levels (**j**), S1PR2 mRNA levels (**k**), S1PR3 protein expression (**l**). Data are means ± SEM of 5–8 mice per group. The densitometric data of the respective bands of interest are normalized by β-actin (lower band). Blots were cropped (black lines) to improve the clarity and conciseness of the presentation. Two-way ANOVA considering stress (S) and Sphk2−/− as independent variables followed by Tukey's post hoc for (**a**–**l**); *S p < 0.05, **S p < 0.01, ***S p < 0.001; #Sphk2−/− p < 0.05; @ (interaction) p < 0.05; ^+^p < 0.05, ^++^p < 0.01 vs Control WT; ^&^p < 0.05 vs Control Sphk2−/−. Two-tailed Student t-test for f; ^%%^p < 0.01. Sphk2: sphingosine kinase 2; WT: wild-type; Sphk2−/−: Sphk2 knockout; S1P: sphingosine-1-phosphate; Sphk1: sphingosine kinase 1; SGPL1: sphingosine-1-phosphate lyase 1; SGPP1: sphingosine-1-phosphate phosphatase 1; SGPP2 sphingosine-1-phosphate phosphatase 2; S1PR1: sphingosine-1-phosphate receptor 1; S1PR2: sphingosine-1-phosphate receptor 2; S1PR3: sphingosine-1-phosphate receptor 3.

General physiological measures were obtained to assess the effects of this stress procedure. Stress caused weight loss (Fig. 1a) and increased plasma corticosterone levels (Fig. 1b) regardless of the genotype. Sphk2−/− mice showed a significantly smaller spleen in both control and stressed mice (Fig. 1c). Thus, stress decreased the weight of animals and increased the plasma corticosterone levels, and Sphk2 deletion only decreased the spleen size.

### Sub-chronic stress caused S1P accumulation in the colon, a decrease in the expression of Sphk2 and its degradation enzymes, and changed S1PR2 and S1PR3 receptor expression

S1P, its synthesis (Sphk1, Sphk2), and degradation (SGPL1, SGPP1, SGPP2) enzymes were measured after stress exposure in wild-type (WT) and Sphk2−/− mice. Stress increased S1P concentration (Fig. 1d) in colon tissue, did not modify Sphk1 mRNA (Fig. 1e), and decreased Sphk2 mRNA (Fig. 1f). Furthermore, stress decreased the transcripts levels of the degradation enzymes SGPL1, SGPP1, and SGPP2 (Fig. 1g–i). Sphk2 deletion downregulated SGPP2 mRNA (Fig. 1i).

At the receptor level, stress exposure did not affect S1PR1 (Fig. 1j) but downregulated S1PR2 mRNA expression (Fig. 1k). A stress effect was detected on S1PR3 mRNA levels (see Supplementary Fig. S1 online), and the protein expression revealed a stress-induced increment (Fig. 1l). No genotype differences were detected for S1P receptors.

Therefore, stress increased S1P and downregulated Sphk2 and its degradation enzymes. Moreover, it upregulated S1PR2 and S1PR3. The only Sphk2 deletion effect was a decrease in SGPP2.

### Sub-chronic stress led to an immune dysregulation towards an inflammatory phenotype at multiple levels, and Sphk2 deletion could boost the T-helper lymphocyte 17 (Th17) response

**Figure 2:**
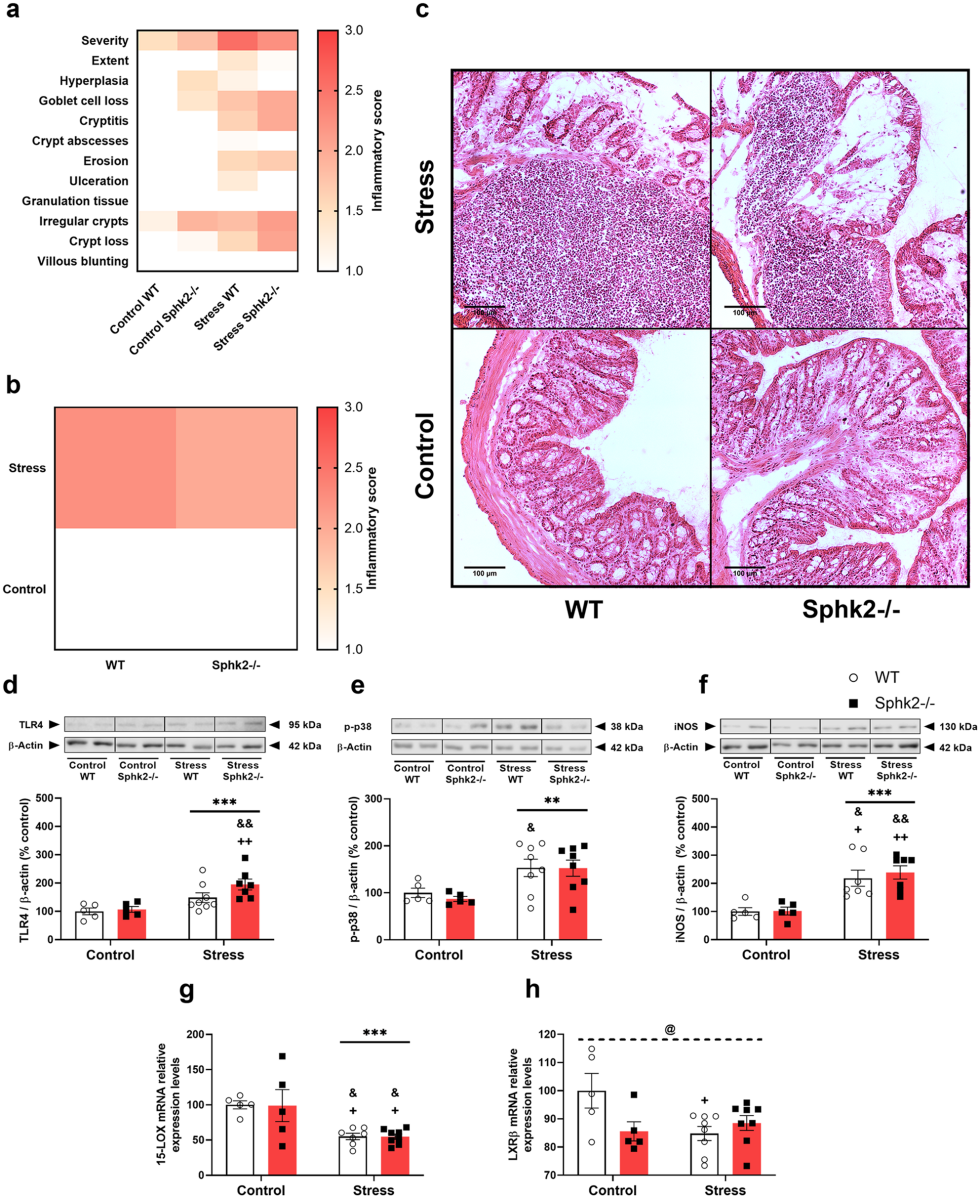
Inflammatory evaluation, TLR4 pathway expression, and anti-inflammatory mechanisms in the colon of WT and Sphk2−/− mice after sub-chronic stress exposure. H&E staining of 10 µm descending colon sections: individual qualitative evaluation (1–4) of parameters related to inflammation: severity, extent, hyperplasia, goblet cell loss, cryptitis, crypt abscesses, erosion, ulceration, granulation tissue, irregular crypts, crypt loss, and villous blunting (**a**), overall inflammatory score (1–4) (**b**), and representative images of each group (**c**), TLR4 protein expression (**d**), p-p38 protein expression (**e**), iNOS protein expression (**f**), LOX-15 mRNA levels (**g**), and LXRβ mRNA levels (**h**). For a and b, data are means of two sections per mice and 5–8 mice per group. For **d**–**h**, data are means ± SEM of 5–8 mice per group. The densitometric data of the respective bands of interest are normalized by β-actin (lower band). Blots were cropped (black lines) to improve the clarity and conciseness of the presentation. Two-way ANOVA considering stress (S) and Sphk2−/− as independent variables followed by Tukey's post hoc; **S p < 0.01, ***S p < 0.001; @ (interaction) p < 0.05; ^+^p < 0.05, ^++^p < 0.01 vs Control WT; ^&^p < 0.05, ^&&^p < 0.01 vs Control Sphk2−/−. Sphk2: sphingosine kinase 2; WT: wild-type; Sphk2−/−: Sphk2 knockout; TLR4: toll-like receptor 4; p-p38: phospho mitogen-activated protein kinase (MAPK) p38; iNOS: inducible nitric oxide synthase; 15-LOX: 15-lipoxygenase; LXRβ: Liver X receptor β.

Hematoxylin & Eosin (H&E) evaluation showed inflammatory features in the colon after stress exposure (Fig. 2a–c). The absence of the Sphk2 gene is associated with architectural distortion of the crypts, which is exacerbated in stressed mice (Fig. 2a). However, it did not have an impact on the overall inflammatory score (Fig. 2b).

Toll-like receptor 4 (TLR4) is a crucial receptor for intestinal immune homeostasis. Stress increased TLR4 protein levels (Fig. 2d) and some of its downstream pro-inflammatory proteins, such as phospho mitogen-activated protein kinase (MAPK) p38 (p-p38) and inducible nitric oxide synthase (iNOS) (Fig. 2e,f). Sphk2 deletion did not modulate the TLR4 pathway. Transcript levels of anti-inflammatory mechanisms, such as 15-lipoxygenase (15-LOX) and liver X receptor β (LXRβ) (Fig. 2g,h) decreased after stress exposure, but no genotype effects were detected. Hence, sub-chronic stress caused inflammation in the colon and Sphk2 deletion did not aggravate it, but Sphk2−/− mice presented architectural abnormalities.

**Figure 3:**
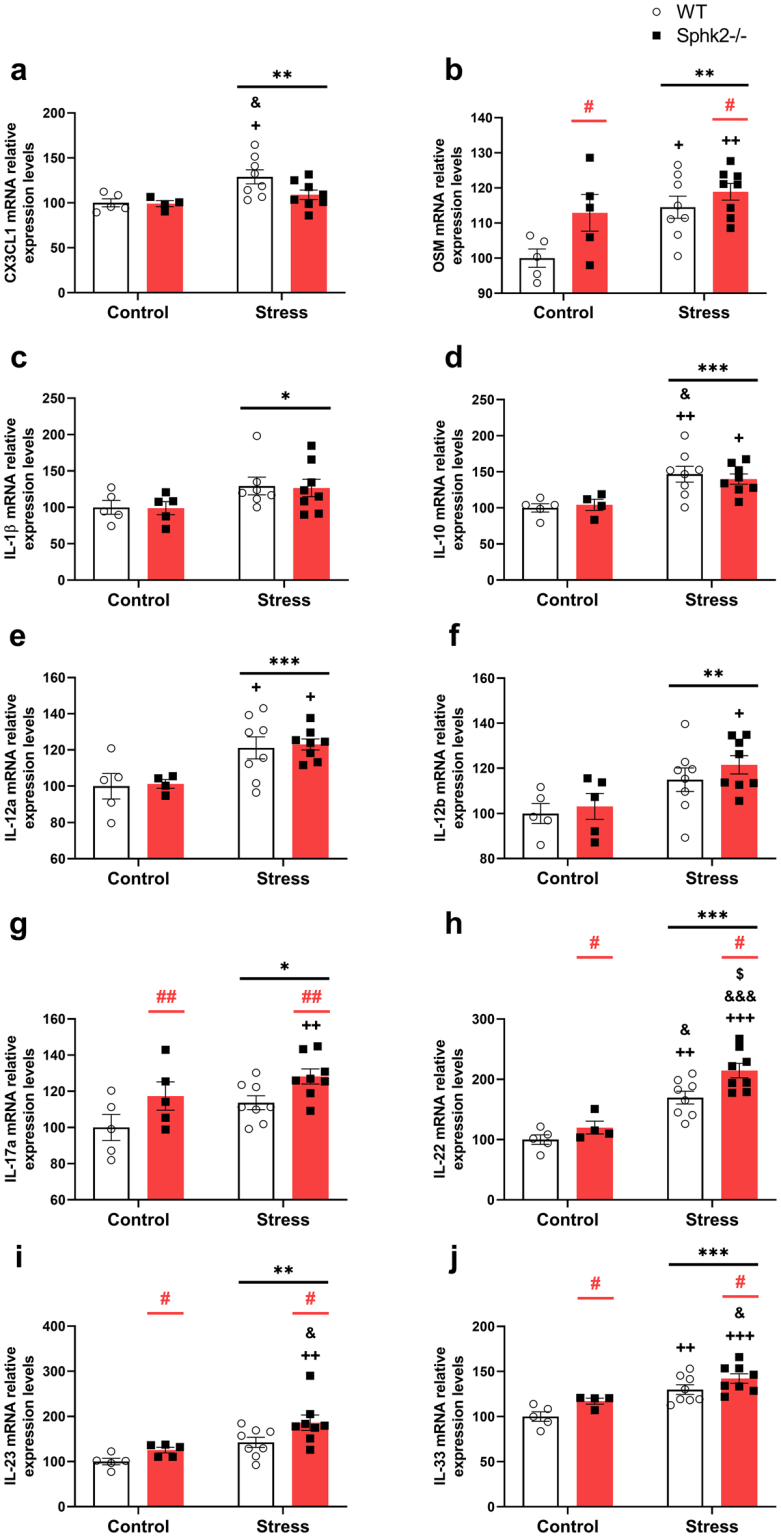
mRNA cytokine expression in the colon of WT and Sphk2−/− mice after sub-chronic stress exposure. mRNA levels of CX3CL1 (**a**), OSM (**b**), IL-1β (**c**) IL-10 (**d**), IL-12a (**e**), IL-12b (**f**), IL-17a (**g**), IL-22 (**h**), IL-23 (**i**), and IL-33 (**j**). Data are means ± SEM of 5–8 mice per group. Two-way ANOVA considering stress (S) and Sphk2−/− as independent variables followed by Tukey's post hoc; *S p < 0.05, **S p < 0.01, ***S p < 0.001; #Sphk2−/− p < 0.05, ##Sphk2−/− p < 0.01; ^+^p < 0.05, ^++^p < 0.01, ^+++^p < 0.001 vs Control WT; ^&^p < 0.05, ^&&&^p < 0.001 vs Control Sphk2−/−; ^$^p < 0.05 vs Stress WT. Sphk2: sphingosine kinase 2; WT: wild-type; Sphk2−/−: Sphk2 knockout; CX3CL1: fractalkine; OSM: oncostatin M; IL: interleukin.

Different cytokines were analyzed to unravel the immune signaling and the possible cellular populations participating in the inflammatory processes. Stress increased the mRNA levels of fractalkine (CX3CL1), oncostatin M (OSM), interleukin (IL)-1β, IL-10, IL-12a, IL-12b, IL-17a, IL-22, IL-23, and IL-33 (Fig. 3a–j). The Sphk2 deletion upregulated the expression of OSM, IL-17a, IL-22, IL-23, and IL-33, reaching the higher levels in the Stress Sphk2−/− experimental group (Fig. 3b,g–j). Thus, the cytokine-expression profile changed towards a Th17 polarization due to stress, which is boosted in Sphk2−/− animals.

**Figure 4:**
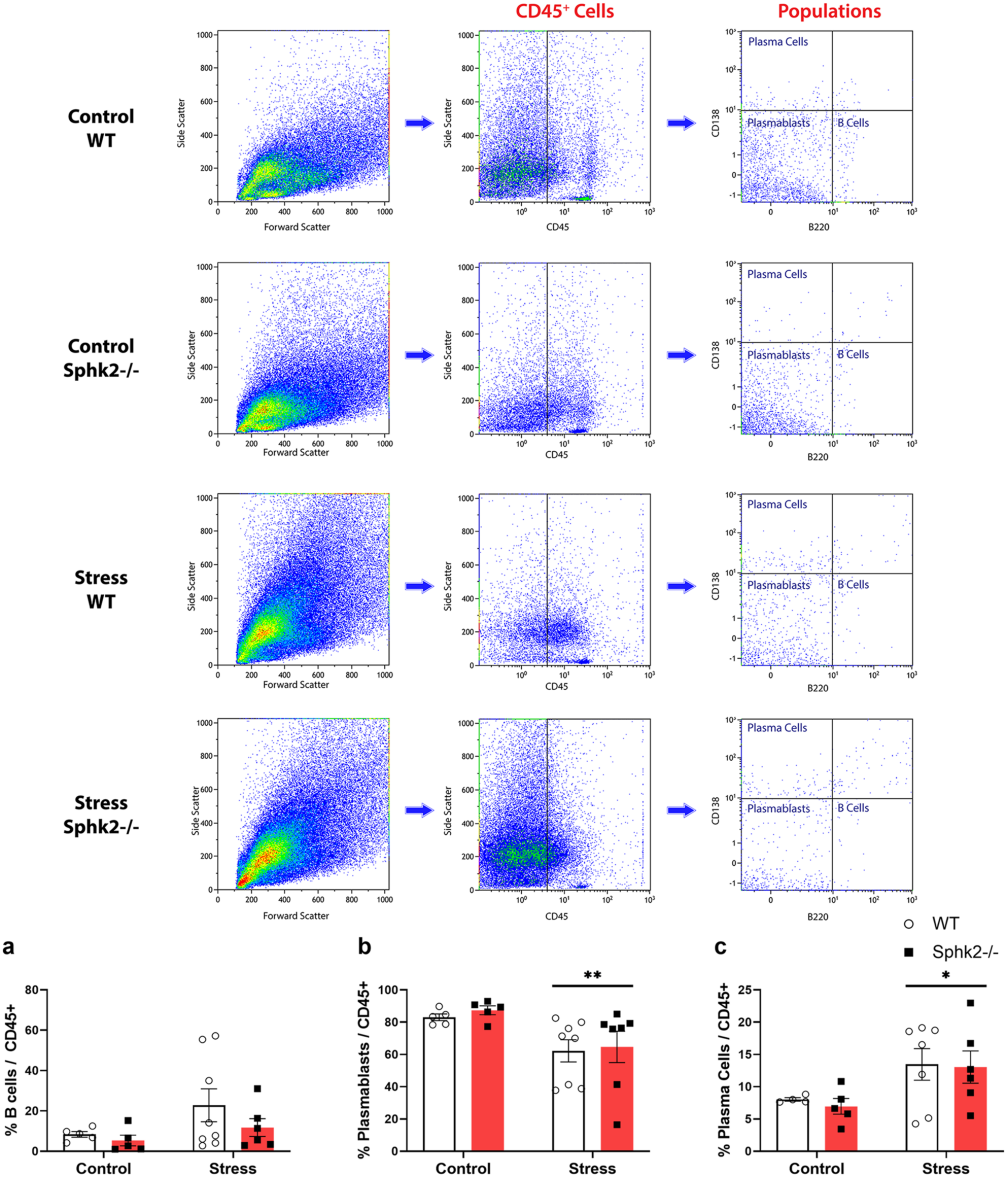
Flow cytometry analysis of B cell differentiation in the colon of WT and Sphk2−/− mice after sub-chronic stress exposure. Representative dot plots showing the gating strategy used to distinguish lymphoid lineage cells (CD45+), B cells (CD45+B220+CD138−/low), plasmablasts (CD45+B220−CD138−/low), and plasma cells (CD45+B220−CD138high). Considering the total number of CD45+ cells as 100%, percentage of B cells (**a**), plasmablasts (**b**), and plasma cells (**c**) were plotted. Data are means ± SEM of 5–8 mice per group. Two-way ANOVA considering stress (S) and Sphk2−/− as independent variables followed by Tukey's post hoc; *S p < 0.05, **S p < 0.01. Sphk2: Sphingosine kinase 2; WT: wild-type; Sphk2−/−: Sphk2 knockout.

**Figure 5:**
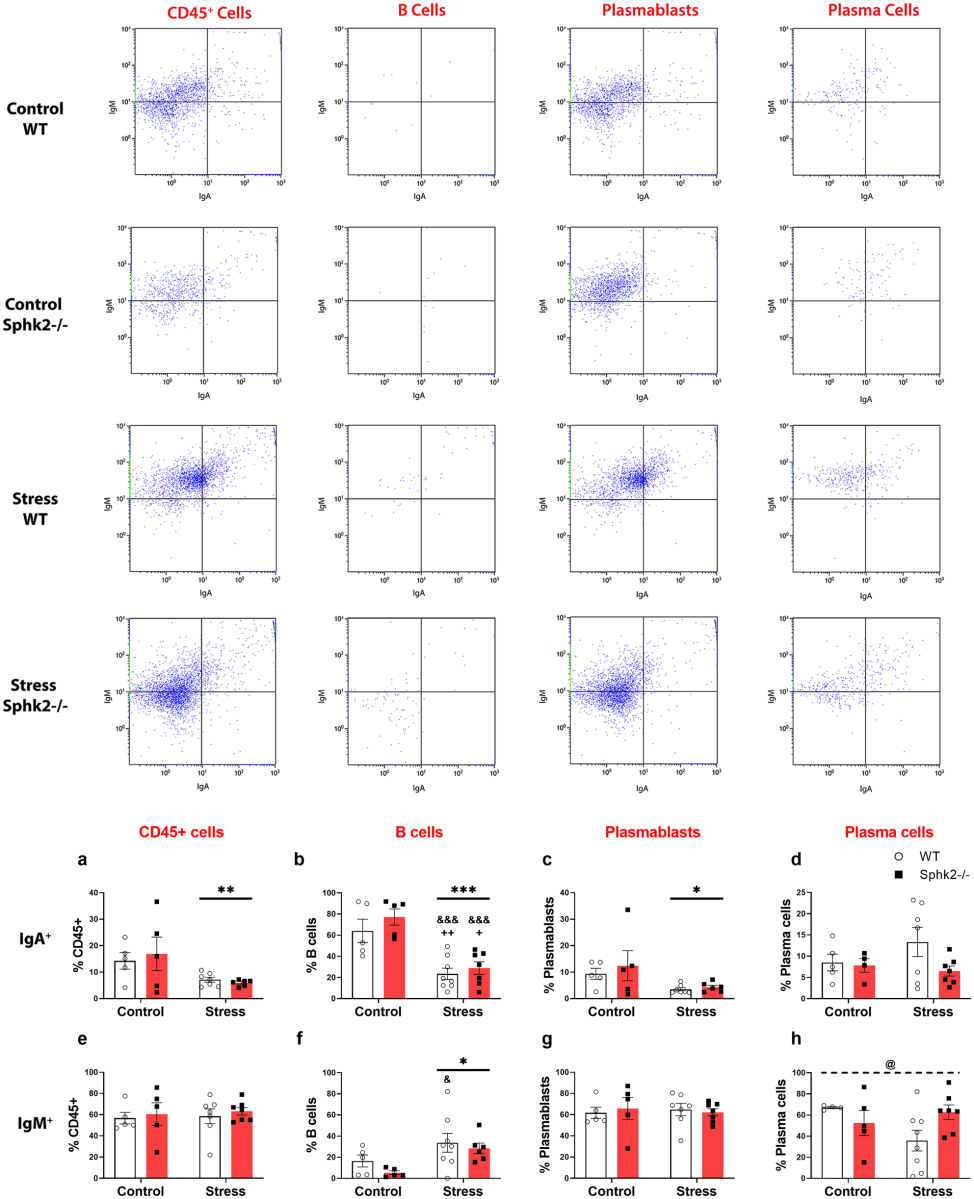
Flow cytometry analysis of IgA and IgM positive cells in different colon immune populations of WT and Sphk2−/− mice after stress exposure. Representative dot plots showing the gating strategy used to distinguish IgA−IgM−, IgA+IgM−, IgA−IgM+, and IgA+IgM+ in each cell population. Percentage of IgA+CD45+ (**a**), IgA+ B cells (**b**), IgA+ plasmablasts (**c**), IgA+ plasma cells (**d**), IgM+CD45+ (**e**), IgM+ B cells (**f**), IgM+ plasmablasts (**g**), IgM+ plasma cells (**h**) were plotted. Data are means ± SEM of 5–8 mice per group. Two-way ANOVA considering stress (S) and Sphk2−/− as independent variables followed by Tukey's post hoc; *S p < 0.05, **S p < 0.01, ***S p < 0.001; @ (interaction) p < 0.05; ^+^p < 0.05, ^++^p < 0.01 vs Control WT; ^&^p < 0.05, ^&&&^p < 0.001 vs Control Sphk2−/−. Sphk2: Sphingosine kinase 2; WT: wild-type; Sphk2−/−: Sphk2 knockout; Ig: immunoglobulin.

Flow cytometry of colon samples was performed to study B cell differentiation and immunoglobulin (Ig)A-IgM production. No significant changes in the percentage of B cells (Fig. 4a) with respect to total lymphoid lineage cells (CD45+) were found, but a decrease in plasmablasts (Fig. 4b) and an increase in plasma cells (Fig. 4c) were observed after stress exposure. Regarding IgA production, stress diminished the global percentage of IgA+CD45+ cells (Fig. 5a). Within the CD45+ population, stress decreased the percentages of IgA+ B cells (Fig. 5b) and IgA+ plasmablasts (Fig. 5c), but did not modify the percentages of IgA+ plasma cells (Fig. 5d). Global percentage of IgM+CD45+ cells did not change under our experimental conditions (Fig. 5e). Considering CD45+ population, stress increased the percentages of IgM+ B cells (Fig. 5f), but did not affect percentages of IgM+ plasmablasts (Fig. 5g). Genotype modified neither IgA nor IgM responses, except for the IgM+ P-cells where there was a significant interaction between variables translated into a value closer to the control group for the Stress Sphk2−/− mice (Fig. 5h). Considering these results, sub-chronic stress affected the B cell differentiation of IgA+ and IgM+ cells but the Sphk2 deletion had no effect on it.

### Sub-chronic stress enhanced intestinal permeability and downregulated some structural proteins, which were differentially expressed in Sphk2−/− mice

**Figure 6:**
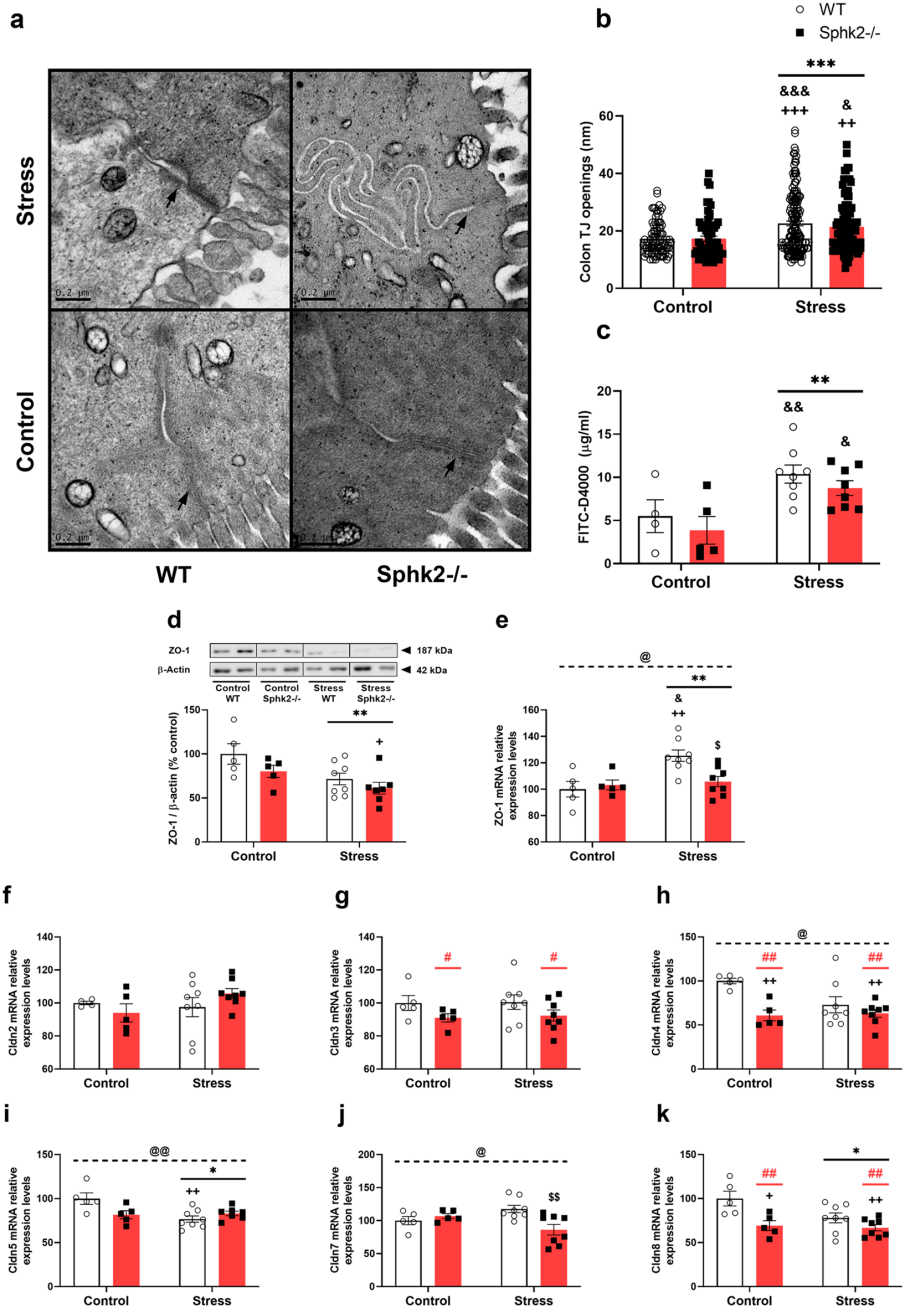
Intestinal permeability analyses and expression levels of structural proteins in the colon of WT and Sphk2−/− mice after sub-chronic stress exposure. Representative transmission electron micrographs of colonic tight junctions (TJs) in epithelial cells of each group (**a**), quantification of TJs (**b**), and FITC-D4000 detected in plasma (**c**), ZO-1 protein expression (**d**), ZO-1 mRNA levels (**e**), Cldn2 mRNA levels (**f**), Cldn3 mRNA levels (**g**), Cldn4 mRNA levels (**h**), Cldn5 mRNA levels (**i**), Cldn7 mRNA levels (**j**), Cldn8 mRNA levels (**k**). For b, data are means ± SEM of 66–168 TJs per group. Arrows indicate TJ ultrastructure. For c-k, data are means ± SEM of 5–8 mice per group. The densitometric data of the respective bands of interest are normalized by β-actin (lower band). Blots were cropped (black lines) to improve the clarity and conciseness of the presentation. Two-way ANOVA considering stress (S) and Sphk2−/− as independent variables followed by Tukey's post hoc; *S p < 0.05, **S p < 0.01, ***S p < 0.001; #Sphk2−/− p < 0.05, ##Sphk2−/− p < 0.01; @ (interaction) p < 0.05, @@ p < 0.01; ^+^p < 0.05, ^++^p < 0.01, ^+++^p < 0.001 vs Control WT; ^&^p < 0.05, ^&&^p < 0.01, ^&&&^p < 0.001 vs Control Sphk2−/−; ^$^p < 0.05, ^$$^p < 0.01 vs Stress WT. Sphk2: sphingosine kinase 2; WT: wild-type; Sphk2−/−: Sphk2 knockout; TJ: tight junction; FITC-D4000: FITC-dextran average molecular weight 4000; ZO-1: zonula occludens-1; Cldn: claudin.

Transmission electron microscopy, plasma presence of fluorescein isothiocyanate-dextran average MW 4000 (FITC-D4000), and structural proteins in the colon were assayed to assess intestinal dysfunction. Stress enhanced intestinal permeability, caused bigger tight junction openings (Fig. 6a,b), and increased FITC-D4000 plasma levels in the stressed groups (Fig. 6c). Sphk2 deletion had no effects on intestinal permeability.

Regarding structural proteins, zonula occludens-1 (ZO-1) expression decreased after stress exposure (Fig. 6d). A stress effect was also detected in ZO-1 mRNA, but there was an interaction between variables with an increase in Stress WT mice in comparison to control and Stress Sphk2−/− groups (Fig. 6e). Claudin (Cldn) family members were analyzed through mRNA expression. Cldn2 levels did not change in our experimental setting (Fig. 6f). Stress decreased Cldn5, 8 (Fig. 6h,i,k). Sphk2−/− mice presented lower levels of Cldn3, 4, 8 (Fig. 6g,h,k). Interactions between stress and genotype were detected for Cldn4, 5, 7 (Fig. 6h–j), showing a decrease in Cldn4 for both Sphk2−/− groups compared to Control WT (Fig. 6h), a downregulation of Cldn5 in Stress WT and a clear trend to decrease in both Sphk2−/− groups (p = 0.05) compared to Control WT (Fig. 6i), and a lowered Cldn7 expression for Stress Sphk2−/− in comparison to Stress WT (Fig. 6j).

Hence, sub-chronic stress increased intestinal permeability but Sphk2 deletion did not affect it. Stress also decreased the expression of several structural proteins in the colon (ZO-1, Cldn5, 8) and Stress Sphk2−/− mice had lower levels of Cldn3, 4, 7, 8.

## Discussion

Our data indicate that sub-chronic stress causes weight loss and a plasma corticosterone increase, two effects typically associated with stress axis activation^15^. The Sphk2 deletion does not affect the general stress response, but these mice have a smaller spleen. Spleen size is controversial since it is not associated with a hyper or hypoactivity. A previous study showed that Sphk2 deletion did not modify spleen lymphocyte number but could decrease CD4+ T-cells after certain immunological challenges^16^. Still, no effects compromising the normal immune system activity were recorded in Sphk2−/− mice beyond mild anaphylaxis with a fast recovery that, a priori, does not impact IBD^17^.

By and large, stress as a trigger of intestinal effects is well-documented^3^, and S1P pathways have been intensely studied in IBD^8,9^. However, to our knowledge, this is the first time showing that stress increases S1P in the colon possibly (and supported by the correlation analyses showed in Supplementary Information online) due to a downregulation of Sphk2 and its degradation enzymes, as it was also described in patients^18^. Our results further support that Sphk2 absence may cause an increase in S1P. Moreover, the SGPP2 downregulation detected in Sphk2−/− mice compared to WT would also contribute to the trend to higher S1P levels under control situations. Other studies^13,19^ pointed to a S1P increase in both colon and plasma of Sphk2−/− animals, possibly due to an Sphk1 potentiation^20^. However, this mechanism did not seem to happen in our experimental setting, at least at a mRNA expression level. Undeniably, further experiments are required to give a full mechanistic explanation of the S1P pathway functioning in the colon after stress exposure.

Sub-chronic stress does not change S1PR1, downregulates S1PR2, and induces S1PR3. Although relevant functions in IBD, only chronic but not acute inflammation affect S1PR1 expression^21^. S1PR2 activity has been associated with Na^+^/K^+^ ATPase inhibition responsible for diarrhea^22^, healthy epithelial barrier function^23^, and suppression of Th17 response^24^. Inflammation^25^ and bacteria^26^ signal through S1PR3 as has been proved in several tissues; however, there is little information about its role in IBD. Considering all this evidence, our results showing a decrease in S1PR2 and an increase in S1PR3 could indicate a deleterious effect in the colon.

S1P accumulation could lead to inflammation, and our results and correlation analyses back up this assertion. Sub-chronic stress causes colon tissue inflammation, activating TLR4 pathways and weakening the expression of some anti-inflammatory molecules. The signature of bacterial TLR4 response appeared in patients with IBD and some polymorphisms were connected to IBD risk^27^. Previous results in our model showed an increase in iNOS expression^15^, and p38 MAPK activity played a role in inflammation-induced colon cancer^28^. Regarding pro-resolving mediators, other studies pointed to a similar decrease in 15-LOX^29^ and LXRβ^30^, in congruence with our results.

The combination of sub-chronic stress and Sphk2 deletion does not lead to a synergistic effect on S1P pathways and inflammation. Most research studies in colon use more aggressive models, such as dextran sodium sulfate (DSS), to evaluate the immune response and the S1P role. Particularly, a study found an increase in colon S1P and inflammatory markers; both exacerbated in Sphk2−/− mice^13^. Our stress protocol is sub-chronic and milder than DSS, being chronic exposure the most connected to pathology development^31^. We hypothesize that the subtle changes caused by a sub-chronic stress exposure could shed light on the stress-induced mechanisms contributing to the worsening of IBD clinical activity but are insufficient to impact the processes controlled by Sphk2 at this inflammatory stage. Undoubtedly, future investigation considering these variables will need to be undertaken. Nevertheless, general H&E evaluation showed changes in some parameters in the Control Sphk2−/− mice compared to Control WT (severity, goblet cell loss, irregular crypts, and crypt loss), which were exacerbated in the Stress Sphk2−/− group. These results and the following related to Th17 response, claudin expression, and crypt architecture, suggest the consequences of Sphk2 absence and S1P involvement in intestinal pathophysiology.

A cytokine panel was performed as an indirect measure of immune signaling towards different immune populations. Our sub-chronic stress protocol induces an increase in CX3CL1, which participates in the recruitment and activation of immune cells, agreeing with previous studies showing an IBD improvement after blocking CX3CL1 signaling^32^. OSM, a pleiotropic cytokine of the IL-6 family recently identified as a biomarker of IBD^33^ and a promoter of intestinal inflammation related to treatment failure^34^, is upregulated after stress exposure. A similar effect is observed for IL-1β, a pro-inflammatory cytokine involved in Th17 response by promoting an IL-17a production, also shown in our experimental setting^35^. Even more, Th17 cells require the IL-6 receptor activity, which can also bind OSM^36^. Up-regulation of IL-12, IL-22, and IL-23 correlate with other IBD models and current therapies against IBD, evincing the importance of Th17 in bowel inflammation^37^. Interestingly, a dual role has been described for IL-33, acting as an alarmin in acute phases but protecting through Foxp3+ Treg cells in chronic stages of the disease. Considering the sub-chronic nature of our stress protocol, the IL-33 increase is consistent with its involvement in the early stages of IBD^38^. This timeframe mimicked by our stress model could explain the IL-10 induction detected. Mice with a defective IL-10 response spontaneously develop IBD over time. However, the anti-inflammatory properties of IL-10 are crucial in the first days after an immunological insult, trying to restore homeostasis. For this reason, other studies also failed to detect acute colon IL-10 variations but showed a decrease at longer times^39^.

Here, the Sphk2 deletion upregulates OSM, IL-17, IL-22, IL-23, and IL-33, with the highest values expressed in the stressed-knockout mice. These results indicate that the Sphk2 absence could interfere with the adaptive immune response in the colon, making mice prone to a more robust Th17 response. Moreover, the positive correlations among the analyzed ILs further point to this Th17 polarization. Some studies connected the S1P signaling with T-cell differentiation^40^, but controversial results about the Sphk2 role in Th17 response have been reported. The use of Sphk2 inhibitors in a psoriasis model blocked the Th17 differentiation^41^, and both Sphk1 and Sphk1/Sphk2 inhibitors attenuated it^42^. However, these studies showed a decrease in S1P levels contrary to the increase detected in the Stress Sphk2−/− group in our model. Indeed, a recent study has described a strong T cell immunopathology in Sphk2−/− mice after challenged with a virus^43^. Considering the relevance of Th17 in IBD, our results suggest further mechanisms that should be considered when studying S1P pathways and possible actions of S1P-based drugs.

IBD research is revisiting B cells due to their critical contribution to immune homeostasis in the colon. Our experimental setting explores B cell differentiation to produce IgA and IgM. Sub-chronic stress causes a drop in plasmablasts and an increase in plasma cells. Focal or diffuse basal plasmacytosis has been recognized as the earliest feature with the highest predictive value for UC diagnosis^44^. Regarding IgA and IgM, stress decreases IgA+CD45+ cells, B cells, and plasmablasts (which could explain the general drop in total plasmablasts) but increases IgM+ B cells. However, the DSS model caused an increment in IgA+ cells and a drop in IgM+^45^, contrary to our results. A feasible explanation relies on the disease stage simulated by our sub-chronic stress model. IgA represents a first defense line against pathogens in the gut, and this stress protocol has reported a decline in its levels^15^. IgM+ cells are crucial in acute immune challenges, and a weakened IgA response together with intestinal permeability could be rooted in the loss of immune tolerance against commensal bacteria. As IBD progresses, uncontrolled inflammation exhausts IgM and induces IgA in response to multiple interactions with bacteria due to tissue damage. Interestingly, CD patients in remission showed elevated IgM+, supporting its role in inflammatory control^46^. Undeniably, more exhaustive studies are mandatory to clarify the role of B cells. Regarding Sphk2 involvement, only an interaction effect between stress and genotype variables but with no significant differences within groups was detected in IgM+ plasma cells. A study described that the pharmacological inhibition of Sphk2 had minimal or no impact on basal levels of circulating human B cells from healthy donors^47^. Other studies pointed to S1P as a regulator of IgA-producing cells^48^, agreeing with the described stress effects. However, Sphk2 deletion did not modify S1P levels in our experimental setting and, therefore, seems not to modify B cell differentiation.

Inflammation and intestinal barrier dysfunction are two phenomena reciprocally regulated. Stress increases intestinal permeability^15^, regardless of the genotype. Nevertheless, another study found an increase in FITC-D4000 permeability in Sphk2−/− mice, suggesting interference with normal epithelial cell growth^49^. Epithelial integrity especially relies on the intercellular junctions between adjacent cells. Considering this and the irregular crypts detected in Sphk2−/− mice, ZO-1 and claudins were analyzed. On the one hand, stress decreases ZO-1 protein expression and Cldn5, 8; thus, impairing colon epithelial function. Up-regulation of ZO-1 mRNA is consistent with our hypothesis of the homeostatic system trying to respond at this sub-chronic stage, but Sphk2−/− mice do not show this recovery effect. On the other hand, Sphk2 deletion is also responsible for lower levels of Cldn3, 4, 8. While Cldn8 expression is restricted to tight junctions, Cldn3, 4, 5, 7 are also located in the lateral plasma membrane^50^. This particular position has been associated with an active role in dynamic remodeling that could be related to the irregular crypts present in Sphk2−/− mice. Interestingly, the significant negative correlations obtained between crypt architecture defects and ZO-1, Cldn4, 8 have also been suggested in the literature^51,52^. Moreover, downregulation of Cldn7 leads to a defective mucosal architecture^53^ and is decreased in Stress Sphk2−/− mice in comparison to Stress WT. Structural abnormalities in the blood–brain barrier of Sphk2 knockout animals further strengthen the idea of the claudin importance for a healthy barrier function^54^. Thus, both sub-chronic stress and Sphk2 deletion modify the expression of structural proteins and claudin differences could be related to the structural abnormalities detected in Sphk2−/− mice.

**Figure 7:**
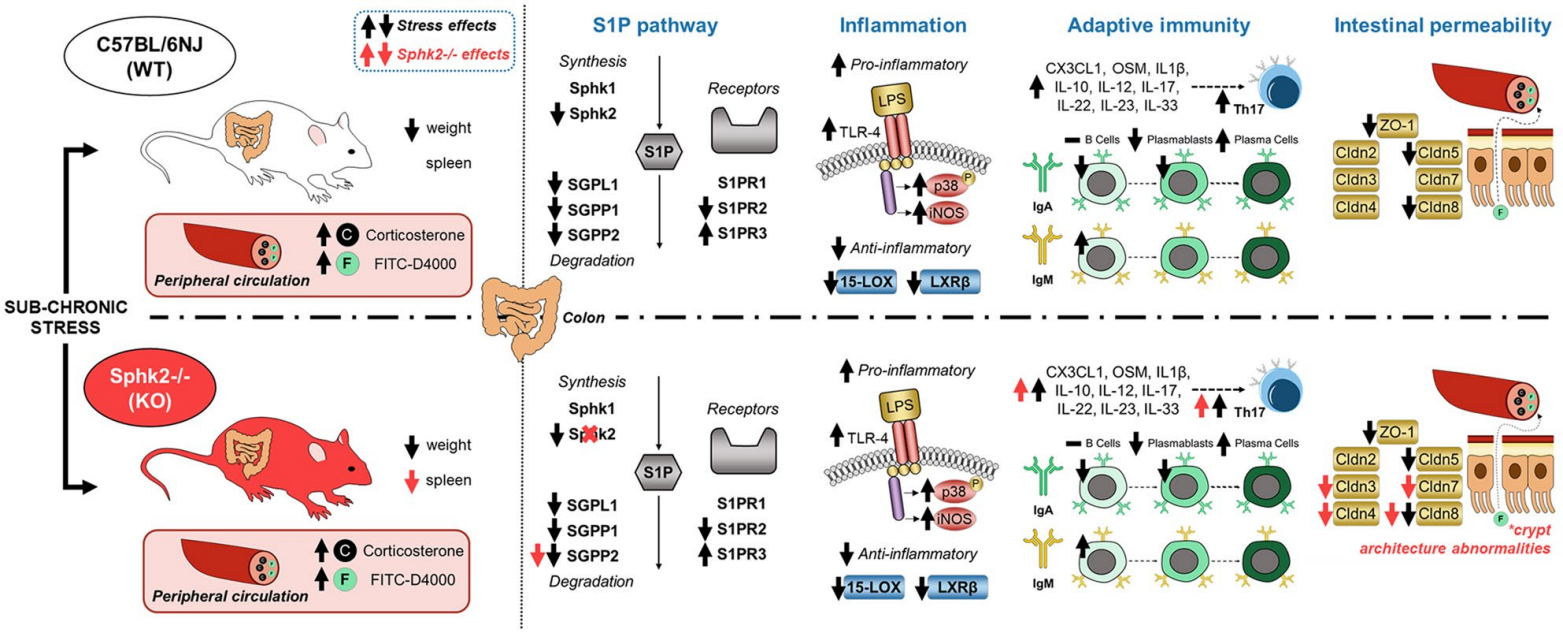
Graphical abstract. The upper part of the image illustrates the sub-chronic stress effects on wild-type (WT) mice. The lower part describes the sub-chronic stress effects on Sphk2 knockout (Sphk2−/−) mice. The black arrows represent the stress effects, and the red arrows the Sphk2 deletion effects. The figure was prepared using the Motifolio Illustration Toolkits (https://motifolio.com) (Motifolio Inc., Ellicott City, MD, USA) and edited with Microsoft PowerPoint 365 (Microsoft Corporation, Redmond, WA, USA). WT: wild-type; Sphk2−/−: Sphk2 knockout; FITC-D4000 (F): fluorescein isothiocyanate-dextran average MW 4,000; S1P: sphingosine-1-phosphate; Sphk1: sphingosine kinase 1; Sphk2: sphingosine kinase 2; SGPL1: sphingosine-1-phosphate lyase 1; SGPP1: sphingosine-1-phosphate phosphatase 1; SGPP2 sphingosine-1-phosphate phosphatase 2; S1PR: sphingosine-1-phosphate receptor; TLR4: Toll-like receptor 4; p-p38: phospho mitogen-activated protein kinase (MAPK) p38; iNOS: inducible nitric oxide synthase; 15-LOX: 15-lipoxygenase; LXRβ: Liver X Receptor β; ZO-1: zonula occludens-1.; Cldn: claudin; CX3CL1: (C-X3-C motif) chemokine ligand 1 (fractalkine); OSM: oncostatin M; IL: interleukin; Th17: T-helper lymphocyte 17; Ig: immunoglobulin.

In a nutshell, our novel findings are summarized and illustrated in Fig. 7.

Taken together, data presented herein reinforce the idea that sub-chronic stress induces colonic inflammation by modulating S1P pathways, dysregulating innate and adaptive immune response, and enhancing intestinal permeability. Sphk2 deletion did not significantly contribute to the stress effects in this model, but the Th17 polarization and crypt architecture abnormalities detected in these animals lay the scene for further investigations. Hence, both stress and S1P can modulate the immune system response in the colon and should be considered in intestinal pathophysiology. A critical limitation of this study hinges on the usage of the Sphk2−/− model. Hematopoietic and extra-hematopoietic sources of S1P seem to have different roles in inflammation, and our model is a germinal knockout unable to analyze these differences that could impair the immune response^55^. Moreover, the size of our control groups could not have enough statistical power to detect relevant differences between WT and KO animals under control situations. Despite these limitations, our research supports that pharmacological modulation of S1P-druggable candidates, including Sphk2 potentiation, represents a promising alternative for future studies.

## Methods

### Animals

Male Sphk2−/− and the suggested C57BL/6NJ control mice (B6N.129S6-Sphk2^tm1Rlp^/J^56^ and C57BL/6NJ, The Jackson Laboratory, ME, USA) initially weighing 23–29 g were housed in groups of 2–3. Animals were maintained under standard temperature and humidity conditions in ventilated racks and in a 12 h light/dark cycle (lights on at 08:00 h) with free access to food and water. Mice were handled daily for 10 days before the beginning of the stress protocol. All experimental procedures adhered to the guidelines of the Animal Welfare Committee of the Universidad Complutense (The ethics committee of Universidad Complutense de Madrid approved the study, ref. PROEX 052/17, April 19th 2017) following European legislation (2010/63/EU), and they were carried out in the Animal facility CAI-UCM. Animal studies are reported in compliance with the ARRIVE guidelines, and all efforts were made to minimize animal suffering and reduce the number of animals.

### Experimental design

Mice from both genotypes were randomly assigned to stress or control groups, and four experimental groups were formed: Control WT (n = 5), Control Sphk2−/− (n = 5), Stress WT (n = 8), and Stress Sphk2−/− (n = 8) (see Supplementary Fig. S2 online). Sphk2 deletion was confirmed by genotyping (see Supplementary Fig. S3 online).

### Stress protocol

A sub-chronic stress mixed model based on immobilization and ultrasound exposure for 2 h/day during four consecutive days was employed^15^ (see Supplementary Fig. S2 online). All experimental procedures started at 11 am to avoid circadian changes affecting the stress response. All the animals were sacrificed at the end of stress exposure using sodium pentobarbital 320 mg/kg i.p. (Vetoquinol, Magny-Vernois, France).

### Preparation of biological samples

Blood samples were obtained by cardiac puncture, anti-coagulated in the presence of ethylenediaminetetraacetic acid (EDTA) 1% w:v, (1 vol EDTA per 50 vol blood), and centrifuged at 1 500 rpm for 15 min to obtain plasma. An aliquot was immediately protected from light and assayed for FITC-D4000 determination. The rest of the plasma was frozen at – 80 °C until assayed.

After blood collection, mice were perfused via ascending aorta with sterile saline solution 0.9% to remove blood from tissues. Descending colon was excised and cut into pieces for the different analyses (see Supplementary Fig. S2 online). For biochemical determinations, samples were frozen at − 80 °C until assayed.

### ELISAs

Corticosterone levels (ENZO Life Sciences, Farmingdale, New York, USA) and S1P (Cloud-Clone Corp., Houston, TX, USA) were measured employing commercially available ELISAs following the manufacturer's instructions.

### Intestinal permeability assay

Mice were fasting for 6 h before the sacrifice, but they had ad libitum access to water. One hour before sacrifice, FITC-D4000 dissolved into drinking water (600 mg/kg) was administered by oral gavage. After obtaining plasma, fluorescence was read (excitation = 485 nm; emission = 528 nm) using a Synergy 2 (BioTek Instruments, Inc., Winooski, VT, USA) spectrophotometer. FITC-D4000 plasma concentration was calculated using a standard curve (100–1.5625 µg/ml).

### H&E staining

Two descending colon fragments of around 0.5 cm per animal were washed gently with phosphate-buffered saline (PBS) 1× pH = 6 and prepared to obtain transverse and longitudinal sections. 10 µm slices were collected using a Leica CM1950 cryostat (Leica, Wetzlar, Germany) and placed on Polysine Microscope Slides (Thermo Fisher Scientific, Waltham, MA, USA). H&E staining procedure consisted of successive incubations following this protocol: tap water 1 min; Mayer’s hematoxylin solution (MilliporeSigma, Burlington, MA, USA) 3 min; tap water 1 min; eosin Y 1% alcoholic (MilliporeSigma) 2 min; Milli-Q water 1 min; ethyl alcohol 50º–70º–96º–100º 5 min each; ethyl alcohol 100º 1 min; Xylol 1 min; Xylol 5 min. DPX Mountant for Histology (MilliporeSigma) was employed for observation in ZEISS Axioplan-2 (Zeiss, Oberkochen, Germany). Three members of the team individually evaluated intestinal inflammation following a guide^57^. The different parameters for each section are the average of the three evaluations, and the final parameter score of each animal is the average of its two different colon sections. The overall inflammatory score of each animal relies on a general evaluation detailed in the guide and based on the individual parameter assessment.

### Flow cytometry

Around 2.5 cm of the descending colon was extracted and washed gently with PBS 1× pH = 6 using a syringe to remove feces. A detailed explanation from tissue to cells is included in Supplementary Information (see Supplementary Methods online). Cells were incubated with anti-CD45-PerCP-Cy5.5 (Ref: 45-0451-82, Thermo Fisher Scientific), anti-CD3-FITC (Ref: 11-0032-82, Thermo Fisher Scientific), anti-B220-PE (Ref: 12–0452-82, Thermo Fisher Scientific), anti-CD138-BV421 (Ref: 142507, Biolegend, San Diego, CA, USA), anti-IgM-PE-Cy7 (Ref: 1140-17, SouthernBiotech, Birmingham, AL, USA), and anti-IgA-APC (Ref: 1165-11, SouthernBiotech), at 1:100 dilution for 30 min at 4 °C light-protected. Samples were washed with 120 μL of Fluorescent Activated Cell Sorting (FACS) buffer and centrifuged (450×*g* for 5 min at 4 °C). Lastly, cells were resuspended in 400 μL of FACS buffer and analyzed using a Gallios flow cytometer (Beckman Coulter, Brea, CA, USA). An appropriate negative control was used to identify each antibody. In these experiments, data were acquired in a mode of 10,000 events. The flow cytometry data were analyzed using Kaluza software (Beckman Coulter). Cell populations were defined as lymphoid lineage cells (CD45+), B cells (CD45+B220+CD138−/low), plasmablasts (CD45+B220−CD138−/low), and plasma cells (CD45+B220−CD138high). Due to an experimental error, one sample from the Stress WT group was lost (n = 7).

### Transmission electron microscopy

Two descending colon fragments of around 0.8 cm per animal—one closer to the cecum and another closer to the rectum—were prepared for transmission electron microscopy analysis following the protocol described in^58^. Sections were examined under a JEOL JEM 1010 microscope (JEOL, Akishima, Tokyo, Japan) at the Spanish National Centre for Electron Microscopy (Madrid, Spain). To evaluate changes in the tight junctions (TJs), the junctional regions of several sections of longitudinally sectioned villi per animal were examined by two team members blinded to the experimental conditions. We analyze 5–15 TJs per section. When possible, we measured the separation between the cell membranes of the adjacent epithelial cells along the length of the TJ complex (0.33 μm approximately) from the apical membrane and reported the maximum distance found between cell membranes for each TJ measured. Quantification was performed using the Image J software (NIH ImageJ, National Biosciences).

### Reverse transcription-quantitative polymerase chain reaction (RT-qPCR)

Colon samples of around 1.5 cm were homogenized in 300 µl of TRIzol reagent (Thermo Fisher Scientific, USA) in the TissueLyser LT (QUIAGEN, Hilden, Germany) for 5 min at 50 s^−1^ and 4 °C. Total cytoplasmic RNA was prepared from samples following the TRIzol datasheet; aliquots were converted to complementary DNA (cDNA) by reverse transcription using random hexamer primers. Semi-quantitative changes in messenger RNA (mRNA) levels were estimated by RT-qPCR using specific conditions: 35–40 cycles of denaturation at 95 °C for 10 s, annealing at 58–65 °C for 15 s depending on the specific set of primers, and extension at 72 °C for 20 s. Reactions were carried out in presence of SYBR green Quantimix Easy Master Mix (Biotools Biotechnological & Medical Laboratories SA Labs, S.A., Madrid, Spain) in a 20 μl reaction in a Rotor-Gene (Corbett Research, Mortlake, NSW, Australia). Primer oligonucleotides for PCR were designed with the Primer3 tool. Target specificity was checked by in silico PCR using the USCS GenomeBrowser and Blast (NCBI) for cDNA and genomic DNA; only primer pairs with no unintended targets were selected (see Supplementary Table S1 online). Relative mRNA concentrations were calculated from the take-off point of reactions using the included software, and Glyceraldehyde-3-Phosphate Dehydrogenase (GAPDH) levels were used as a housekeeper.

### Western Blot

Proteins were extracted from colon tissues of around 1.5 cm, adjusted using the Bradford method and Western Blotted as previously described^15^. Specific primary antibodies were: S1PR3 (Item No. 10006373, Cayman Chemical, Ann Arbor, Michigan, USA, 1:1000); TLR4 (sc-293072, Santa Cruz Biotechnology, Dallas, TX, USA, 1:1000); p-p38 (sc-17852R, Santa Cruz Biotechnology, 1:1000); iNOS (ab15323, Abcam, Cambridge, UK, 1:1000); ZO-1 (ab216880, Abcam, 1:1000). The blots were cut prior to hybridization with antibodies due to the limited amount of protein extract available. Blots were imaged using an Odyssey Fc System (Li-COR Biosciences, Lincoln, NE, USA) and quantified by densitometry using ImageJ software (NIH, Bethesda, MD, USA). All densitometries were obtained in arbitrary units of optical density and expressed as a percentage of the control group (100%). Several exposition times were analyzed to ensure the linearity of the band intensities. Loading control (blots shown in the respective figures) was β-actin (A5441, MilliporeSigma). All full-length data of western blots are included in Supplementary Information (see Supplementary Figs. S4, S5, S6, S7, S8 online).

### Statistical analyses

Data in text and figures are expressed as mean ± standard error of the mean (SEM). The Grubbs' test was performed to identify outliers. Considering stress and Sphk2−/− as independent variables, a two-way analysis of variance (ANOVA) was used, followed by Tukey's post hoc test for multiple comparisons. All the results of the ANOVA analyses (F values and dfs) are included in Table 1. For the particular case of Sphk2 mRNA analysis, a two-tailed Student t-test between Control WT and Stress WT was performed. A p-value < 0.05 was considered statistically significant. Data were analyzed using GraphPad Prism 8 (GraphPad Software, San Diego, CA, USA). To further support manuscript discussion, Pearson's correlations between several parameters were analyzed (see Supplementary Figs. S9, S10, S11 and Supplementary Tables S2, S3, S4 online).

**Table 1:** Two-way ANOVA analyses (F values and dfs).

Parameter	Stress	Sphk2−/−	Interaction
%weight increase	**F**_**(1,21)**_** = 10.83; p = 0.0035**	F_(1,21)_ = 0.4230; p = 0.5225	F_(1,21)_ = 0.4073; p = 0.5303
Corticosterone	**F**_**(1,21)**_** = 9.982; p = 0.0047**	F_(1,21)_ = 0.08156; p = 0.7780	F_(1,21)_ = 1.301; p = 0.2668
%spleen weight	F_(1,22)_ = 0.2001; p = 0.6590	**F**_**(1,22)**_** = 6.580; p = 0.0177**	F_(1,22)_ = 0.06906; p = 0.7952
S1P	**F**_**(1,21)**_** = 8.996; p = 0.0068**	F_(1,21)_ = 1.151; p = 0.2955	F_(1,21)_ = 0.1854; p = 0.6711
Sphk1	F_(1,22)_ = 1.654; p = 0.2118	F_(1,22)_ = 0.2767; p = 0.6041	F_(1,22)_ = 0.1868; p = 0.6698
Sphk2	-	-	-
SGPL1	**F**_**(1,22)**_** = 5.243; p = 0.0320**	F_(1,22)_ = 2.132; p = 0.1584	F_(1,22)_ = 2.012e-008; p = 0.9999
SGPP1	**F**_**(1,22)**_** = 11.35; p = 0.0028**	F_(1,22)_ = 2.572; p = 0.1230	F_(1,22)_ = 0.5859; p = 0.4521
SGPP2	F_(1,22)_ = 1.797; p = 0.1937	**F**_**(1,22)**_** = 6.766; p = 0.0163**	**F**_**(1,22)**_** = 7.396; p = 0.0125**
S1PR1	F_(1,22)_ = 1.699; p = 0.2059	F_(1,22)_ = 0.7525; p = 0.3951	F_(1,22)_ = 3.340; p = 0.0812
S1PR2	**F**_**(1,22)**_** = 4.630; p = 0.0427**	F_(1,22)_ = 0.04937; p = 0.8262	F_(1,22)_ = 0.02840; p = 0.8677
S1PR3	**F**_**(1,20)**_** = 6.417; p = 0.0198**	F_(1,20)_ = 0.002192; p = 0.9631	**F**_**(1,20)**_** = 1.211; p = 0.2843**
TLR4	**F**_**(1,21)**_** = 15.89; p = 0.0007**	F_(1,21)_ = 2.439; p = 0.1333	F_(1,21)_ = 1.328; p = 0.2621
p-p38	**F**_**(1,22)**_** = 12.47; p = 0.0019**	F_(1,22)_ = 0.1634; p = 0.6900	F_(1,22)_ = 0.1343; p = 0.7175
INOS	**F**_**(1,20)**_** = 29.29; p < 0.0001**	F_(1,20)_ = 0.2215; p = 0.6430	F_(1,20)_ = 0.1586; p = 0.6947
15-LOX	**F**_**(1,21)**_** = 19.68; p = 0.0002**	F_(1,21)_ = 0.004723; p = 0.9459	F_(1,21)_ = 0.001169; p = 0.9730
LXRβ	F_(1,22)_ = 2.936; p = 0.1007	F_(1,22)_ = 2.237; p = 0.1490	**F**_**(1,22)**_** = 6.441; p = 0.0187**
CX3CL1	**F**_**(1,21)**_** = 8.249; p = 0.0091**	F_(1,21)_ = 2.375; p = 0.1382	F_(1,21)_ = 1.961; p = 0.1760
OSM	**F**_**(1,22)**_** = 9.218; p = 0.0061**	**F**_**(1,22)**_** = 6.576; p = 0.0177**	F_(1,22)_ = 1.595; p = 0.2199
IL-1β	**F**_**(1,21)**_** = 5.808; p = 0.0252**	F_(1,21)_ = 0.02637; p = 0.8725	F_(1,21)_ = 0.004779; p = 0.9455
IL-10	**F**_**(1,21)**_** = 18.70; p = 0.0003**	F_(1,21)_ = 0.01580; p = 0.9012	F_(1,21)_ = 0.3324; p = 0.5703
IL-12a	**F**_**(1,21)**_** = 15.27; p = 0.0008**	F_(1,21)_ = 0.07968; p = 0.7805	F_(1,21)_ = 0.004024; p = 0.9500
IL-12b	**F**_**(1,22)**_** = 10.71; p = 0.0035**	F_(1,22)_ = 0.9311; p = 0.3451	F_(1,22)_ = 0.1147; p = 0.7380
IL-17a	**F**_**(1,22)**_** = 5.055; p = 0.0349**	**F**_**(1,22)**_** = 8.498; p = 0.0080**	F_(1,22)_ = 0.06996; p = 0.7939
IL-22	**F**_**(1,21)**_** = 47.77; p < 0.0001**	**F**_**(1,21)**_** = 7.460; p = 0.0125**	F_(1,21)_ = 1.114; p = 0.3033
IL-23	**F**_**(1,22)**_** = 14.33; p = 0.0010**	**F**_**(1,22)**_** = 6.424; p = 0.0189**	F_(1,22)_ = 0.4412; p = 0.5135
IL-33	**F**_**(1,21)**_** = 23.65; p < 0.0001**	**F**_**(1,21)**_** = 6.719; p = 0.0170**	F_(1,21)_ = 0.1867; p = 0.6700
% B cells/CD45+	F_(1,20)_ = 2.801; p = 0.1098	F_(1,20)_ = 1.281; p = 0.2711	F_(1,20)_ = 0.4127; p = 0.5279
% plasmablasts/CD45+	**F**_**(1,21)**_** = 8.670; p = 0.0077**	F_(1,21)_ = 0.2110; p = 0.6507	F_(1,21)_ = 0.01597; p = 0.9006
% Plasma cells/CD45+	**F**_**(1,18)**_** = 6.700; p = 0.0185**	F_(1,18)_ = 0.1133; p = 0.7403	F_(1,18)_ = 0.02198; p = 0.8838
% IgA+CD45+	**F**_**(1,19)**_** = 8.371; p = 0.0093**	F_(1,19_) = 0.05343; p = 0.8197	F_(1,19)_ = 0.3688; p = 0.5508
% IgA+ B cells	**F**_**(1,21)**_** = 36.80; p < 0.0001**	F_(1,21)_ = 1.630; p = 0.2156	F_(1,21)_ = 0.2720; p = 0.6074
% IgA+ plasmablasts	**F**_**(1,19)**_** = 6.833; p = 0.0171**	F_(1,19)_ = 0.5317; p = 0.4748	F_(1,19)_ = 0.1750; p = 0.6804
% IgA+ plasma cells	F_(1,19)_ = 0.4779; p = 0.4977	F_(1,19)_ = 2.227; p = 0.1521	F_(1,19)_ = 1.493; p = 0.2367
% IgM+CD45+	F_(1,20)_ = 0.1023; p = 0.7524	F_(1,20)_ = 0.3953; p = 0.5367	F_(1,20)_ = 0.004599; p = 0.9466
% IgM+ B cells	**F**_**(1,20)**_** = 7.883; p = 0.0109**	F_(1,20)_ = 1.312; P = 0.2655	F_(1,20)_ = 0.1618; P = 0.6917
% IgM+ plasmablasts	F_(1,20)_ = 0.002136; p = 0.9636	F_(1,20)_ = 0.01444; P = 0.9055	F_(1,20)_ = 0.2722; P = 0.6076
% IgM+ plasma cells	F_(1,20)_ = 1.212; p = 0.2840	F_(1,20)_ = 0.4073; p = 0.5306	**F**_**(1,20)**_** = 4.699; p = 0.0424**
Colon TJ openings	**F**_**(1,452)**_** = 28.96; p < 0.0001**	F_(1,452)_ = 0.4204; p = 0.5171	F_(1,452)_ = 0.4936; p = 0.4827
FITC-D4000	**F**_**(1,21)**_** = 14.57; p = 0.0010**	F_(1,21)_ = 1.622; p = 0.2167	F_(1,21)_ = 5.075e-005; p = 0.9944
ZO-1 WB	**F**_**(1,21)**_** = 8.886; p = 0.0071**	F_(1,21)_ = 3.570; p = 0.0727	F_(1,21)_ = 0.3328; p = 0.5701
ZO-1 mRNA	**F**_**(1,22)**_** = 9.261; p = 0.0060**	F_(1,22)_ = 3.167; p = 0.0889	**F**_**(1,22)**_** = 6.183; p = 0.0210**
Cldn2	F_(1,21)_ = 0.8661; p = 0.3626	F_(1,21)_ = 0.05279; p = 0.8205	F_(1,21)_ = 2.044; p = 0.1676
Cldn3	F_(1,22)_ = 0.05430; p = 0.8179	**F**_**(1,22)**_** = 4.363; p = 0.0485**	F_(1,22)_ = 0.01108; p = 0.9171
Cldn4	F_(1,22)_ = 3.001; p = 0.0972	**F**_**(1,22)**_** = 11.86; p = 0.0023**	**F**_**(1,22)**_** = 4.325; p = 0.0494**
Cldn5	**F**_**(1,21)**_** = 6.550; p = 0.0183**	F_(1,21)_ = 1.941; p = 0.1781	**F**_**(1,21)**_** = 8.442; p = 0.0085**
Cldn7	F_(1,22)_ = 0.04552;p = 0.8330	**F**_**(1,22)**_** = 3.199; p = 0.0875**	**F**_**(1,22)**_** = 7.814; p = 0.0105**
Cldn8	**F**_**(1,22)**_** = 4.448; p = 0.0466**	**F**_**(1,22)**_** = 13.16; p = 0.0015**	F_(1,22)_ = 2.838; p = 0.1062

Statistically significant values (p < 0.05) are in bold.

### Ethics declarations

All experimental procedures adhered to the guidelines of the Animal Welfare Committee of the Universidad Complutense (PROEX052/17) following European legislation (2010/63/EU), and they were carried out in the Animal facility CAI-UCM. Animal studies are reported in compliance with the ARRIVE guidelines, and all efforts were made to minimize animal suffering and reduce the number of animals.

## Supplementary Information

Supplementary Information.

## Data Availability

The datasets generated during and/or analysed during the current study are available from the corresponding author on reasonable request.
