# Supplementary material for: Sphk2 deletion is involved in structural abnormalities and Th17 response but does not aggravate colon inflammation induced by sub-chronic stress
Source: Sci Rep. 2022 Mar 8;12:4073. doi: 10.1038/s41598-022-08011-8 (PMC8904788; doi:10.1038/s41598-022-08011-8)
Supplement: Supplementary file 1 — Supplementary Information. [file 41598_2022_8011_MOESM1_ESM.pdf]

SUPPLEMENTARY INFORMATION

Sphk2 deletion is involved in structural abnormalities and Th17 response but does not aggravate colon inflammation induced by sub-chronic stress.

Scientific Reports

David Martín-Hernández*, Irene L. Gutiérrez, Marta González-Prieto, Karina S. MacDowell, Javier Robledo-Montaña, Hiram Tendilla-Beltrán, Natalia Calleja-Rodríguez, Álvaro G Bris, Cristina Ulecia-Morón, Beatriz Moreno, Javier R. Caso, Borja García-Bueno, Sandra Rodrigues-Mascarenhas, Ignacio Marín-Jiménez, Juan Carlos Leza, Luis Menchén

Correspondence (*): Dr. David Martín Hernández

Servicio de Psiquiatría del Niño y del Adolescente, Instituto de Psiquiatría y Salud Mental. Hospital General Universitario Gregorio Marañón.

e-mail: davidmhbiotec@gmail.com

- Supplementary Fig.S1: S1PR3 mRNA levels
- Supplementary Fig.S2: Experimental design, stress protocol, and sample collection
- Supplementary Fig.S3: Genotyping
- Supplementary Methods: Flow cytometry details
- Supplementary Table S1: Primer sequences for RT-qPCR
- Supplementary Fig.S4, S5, S6, S7, S8: Full-length data of western blots
- Supplementary Methods: Statistical analyses - correlation analyses
    - Supplementary Fig.S9 and Table S2: Correlations of S1P pathways
    - Supplementary Fig.S10 and Table S3: Correlations of S1P/Sphk2 with inflammatory parameters
    - Supplementary Fig.S11 and Table S4: Correlations of S1P/Sphk2 with structural proteins and crypt architecture abnormalities

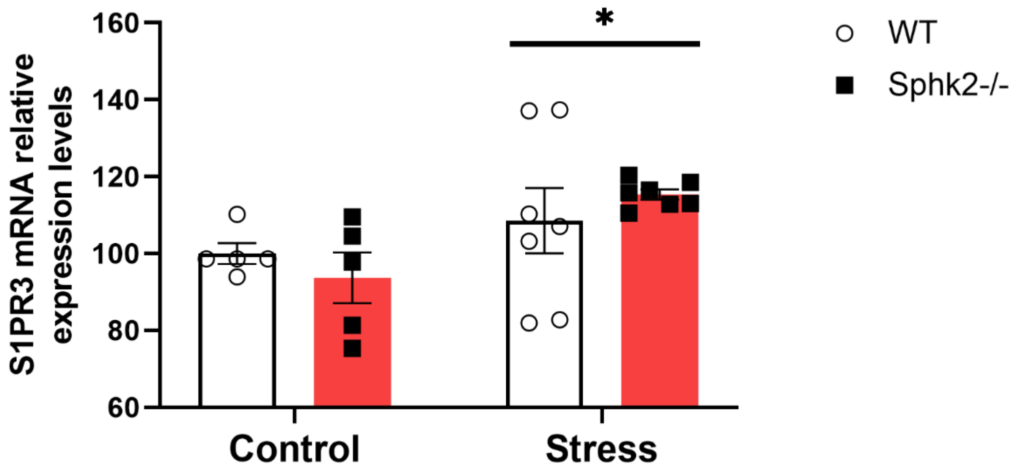

Supplementary Fig.S1 S1PR3 mRNA levels. Data are means ± SEM of 5-8 mice per group. Two-way ANOVA considering stress (S) and Sphk2-/- as independent variables followed by Tukey's post hoc. Stress: $F_{(1,20)}$=6.417; p=0.0198 (*). Sphk2-/-: $F_{(1,20)}$=0.002192; p=0.9631. Interaction: $F_{(1,20)}$=1,211; p=0.2843

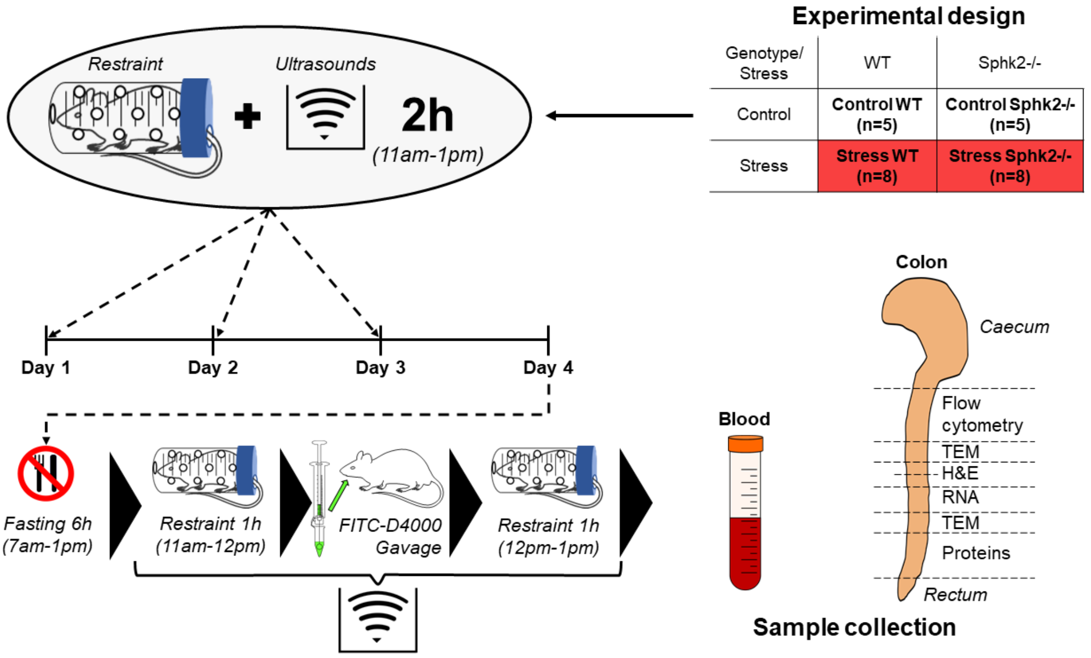

Supplementary Fig.S2 Experimental design, stress protocol, and sample collection**.** Mice were placed in 50 mL Falcon Conical Centrifuge Tubes (Thermo Fisher Scientific, MA, USA) with multiple ventilation holes, one of them in the cap to accommodate the mouse's tail. The tubes were 11.5 cm in length, not allowing forward, backward, or rotational movement. Acoustic stress was induced using an Ultrasons-HD 40 kHz bath (JP Selecta, Spain). All experimental procedures started at 11 am to avoid circadian changes affecting the stress response. On the last day of stress, mice were quickly freed at 12 pm to administer FITC-dextran average molecular weight 4 000 (FITC-D4000) by gavage (see intestinal permeability assay section) and returned to the restraint device. All the animals were sacrificed at the end of stress exposure using sodium pentobarbital 320 mg/kg i.p. (Vetoquinol, France). The figure was prepared using the Motifolio Illustration Toolkits (https://motifolio.com) (Motifolio Inc., Ellicott City, MD, USA) and edited with Microsoft PowerPoint 365 (Microsoft Corporation, Redmond, WA, USA). FITC-D4000: FITC-dextran average molecular weight 4,000; Sphk2: sphingosine kinase 2; WT: wild type; Sphk2-/-: Sphk2 knockout; TEM: transmission electron microscopy; H&E: hematoxylin & eosin

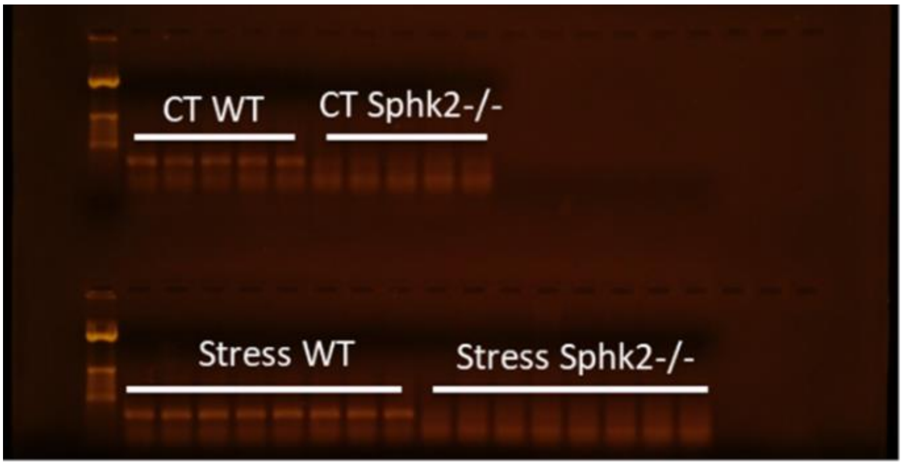

Supplementary Fig.S3 Genotyping of control wild-type (CT WT), control Sphk2-/- (CT Sphk2-/-), Stress WT, and Stress Sphk2-/- mice. The visible bands represent the presence of Sphk2 gene in the samples after PCR amplification. Tail sections (<1 cm) were homogenized in Tail Buffer (Tris 1M, EDTA 5 mM, Tween 10%) at 55 ºC overnight, incubated at 100 ºC for 10 min, and centrifuged at 1300 rpm and room temperature for 10 min to obtain DNA. DNA samples were amplified by PCR using specific Sphk2 primers (forward: 5’-AGAACGACAGAACCATGCCC-3’ , and reverse: 5’-CAGTCTGGCCGATCAAGGAG-3’). 20 µl of the PCR product were mixed with 2.4 µl of load buffer and size-separated in an agarose-TBE 1X gel with ethidium bromide (100 V). Bands were made visible under UV exposure

Supplementary Methods Flow cytometry details

Tissue was placed in 10 mL of cold cell dissociation buffer [Hank's balanced salt solution without Ca2+ and Mg2+ plus 4-(2-hydroxyethyl)-1-piperazineethanesulfonic acid (HEPES) 10 mM, and EDTA 5mM]. The tubes were placed at 37 ºC for 15 min, inverting them every 5 minutes. After a rigorous vortex, the gut segments were incubated at 37 ºC for 10 min in 2.5 mL cell dissociation buffer, overturning them every 5 min, and vortexed. After repeating both cell dissociation buffer steps, the tissues were rinsed with PBS 1x. The excess liquid was removed with tissue paper. The colon samples were minced with a blade until no large clumps were found and subsequently incubated at 37 ºC for 15 min in 1.25 mL of ice-cold Digestion Mix [Hank's balanced salt solution plus dispase II 0.5 U/mL, collagenase D 0.5 mg/mL, fetal bovine serum 2%, and DNAse I 10 U/mL], inverting the tubes every 5 min. After rigorous vortex, 1.25 mL of fresh Digestion Mix was added to each sample and incubated at 37 ºC for 15 min, inverting the tubes every 5 min, and vortexing afterward. The suspension was filtered to a 50 mL tube through a 70 µm strainer, previously wet with neutralization medium [Roswell Park Memorial Institute (RPMI) 1640 plus 2% of Penicillin/Streptomycin 50× and 10% of fetal bovine serum]. The enzymes were neutralized with 3.5 mL of neutralization medium. Immediately, scattered cells were rinsed with 2 mL of the same medium. Samples were centrifuged at 700 x g for 10 min at room temperature, resuspended in 10 mL of PBS 1x, and then centrifuged at 700 x g for 10 min at room temperature. Cells were resuspended in 70 µL of Fluorescent Activated Cell Sorting (FACS) buffer [(PBS 1x plus fetal bovine serum 2%), EDTA 5mM, sodium azide 0.02%] and incubated with 20 µL of Fc-Block solution [Fluorescent Activated Cell Sorting buffer plus Fc 0.02 mg/mL] for 10 min at 4 ºC. Cells were incubated with anti-CD45-PerCP-Cy5.5 (Ref: 45-0451-82, Thermo Fisher Scientific, Waltham, MA, USA), anti-CD3-FITC (Ref: 11-0032-82, Thermo Fisher Scientific), anti-B220-PE (Ref: 12-0452-82, Thermo Fisher Scientific), anti-CD138-BV421 (Ref: 142507, Biolegend, San Diego, CA, USA), anti-IgM-PE-Cy7 (Ref: 1140-17,

SouthernBiotech, Birmingham, AL, USA), and anti-IgA-APC (Ref: 1165-11, SouthernBiotech), at 1:100 dilution for 30 min at 4 °C light-protected. Samples were washed with 120 µL of Fluorescent Activated Cell Sorting buffer and centrifuged (450 x g for 5 min at 4 °C). Lastly, cells were resuspended in 400 µL of FACS buffer and analyzed using a Gallios flow cytometer (Beckman Coulter, Brea, CA, USA). An appropriate negative control was used to identify each antibody. In these experiments, data were acquired in a mode of 10,000 events. The flow cytometry data were analyzed using Kaluza software (Beckman Coulter). Cell populations were defined as lymphoid lineage cells (CD45+), B cells (CD45+B220+CD138-/low), plasmablasts (CD45+B220-CD138-/low), and plasma cells (CD45+B220-CD138high).

TARGET	FORWARD	REVERSE
**Sphk1**	CTGGTGTGTGCAGAGGAGTT	GGAGGCTACACAGGGGTTTC
**Sphk2**	AGAACGACAGAACCATGCCC	CAGTCTGGCCGATCAAGGAG
**SGPL1**	TTCCGGGTGAAAGGTGTGAC	GCCAGTCTGCACCAACAAAG
**SGPP1**	CATCGCCATGTTCCTGCTCA	GGCAAACTAGTGAACTCCAGC
**SGPP2**	CCTGATGATGGCTGTGGTGT	TCCAGGGAATCGATGAGGGT
**S1PR1**	CACCCACTACCCCAGCATTT	GCACATTCTCCCTTCCCTCC
**S1PR2**	GTGGCTCTGTACGTCCGAAT	ATGGTGACCGTCTTGAGCAG
**S1PR3**	GGAGAGAAACCTGAGGCCAC	GACTGCGGGAAGAGTGTTGA
**CX3CL1**	CGGTGCTATCTGCCCTATGT	CGGAGAGCTCCAGAAAACAC
**OSM**	CCAGAGTACCAGGACCCAGT	AGAGGAAGAGTTGGAGCAGC
**IL-1β**	TGGAGAGTGTGGATCCCAAGCAAT	TGCTTGTGAGGTGCTGATGTACCA
**IL-10**	GCATGGCCCAGAAATCAAGG	TCACTCTTCACCTGCTCCAC
**IL-12a**	CATCCAGCAGCTCCTCTCAG	TCTTCAGCAGGTTTCGGGAC
**IL-12b**	TGAAGGAGACAGAGGAGGGG	GAACACATGCCCACTTGCTG
**IL-17a**	CCCTCAGACTACCTCAACCG	GCTCTCAGGCTCCCTCTTCA
**IL-22**	TCCAGCAGCCATACATCGTC	TCCAGGGTGAAGTTGAGCAC
**IL-23**	AATGTGCCCCGTATCCAGTG	GGAGGTGTGAAGTTGCTCCA
**IL-33**	ATGAGTCTCCCTGTCCTGCA	TGTGAAGGACGAAGAAGGCC
**15-LOX**	GGGACAATGGACACCGTTATTA	CCAGGTACTGCTGACTACAAAG
**LXR-β**	ATCTTTCTCCGACCAGCCCA	CCACAATCTCCTGGACCGAG
**ZO-1**	TACCAAACCACAGCCTCCAC	CAGCAAGAGAAGCACCTGGA
**Cldn2**	GTTCGCCTTTCTCTGGACCT	AGTGTCTCTGGCAAGCTGAC
**Cldn3**	GAGTGCTTTTCCTGTTGGCG	CGTACAACCCAGCTCCCATC
**Cldn4**	AGCAACGACAAGCCCTACTC	TCCCCAGCAAGCAGTTAGTG
**Cldn5**	GTTAAGGCACGGGTAGCACT	GTACTTCTGTGACACCGGCA
**Cldn7**	AAAGCGAAGAAGGCCCGAAT	CCTGCCCAGCCGATAAAGAT
**Cldn8**	CTGGAGGAGCACTGTTCTGT	GTTGAGTGGTGCGATGGGAT
**GAPDH**	TGCACCACCAACTGCTTAGC	GGCATGGACTGTGGTCATGAG

Supplementary Table S1 Primer sequences for RT-qPCR

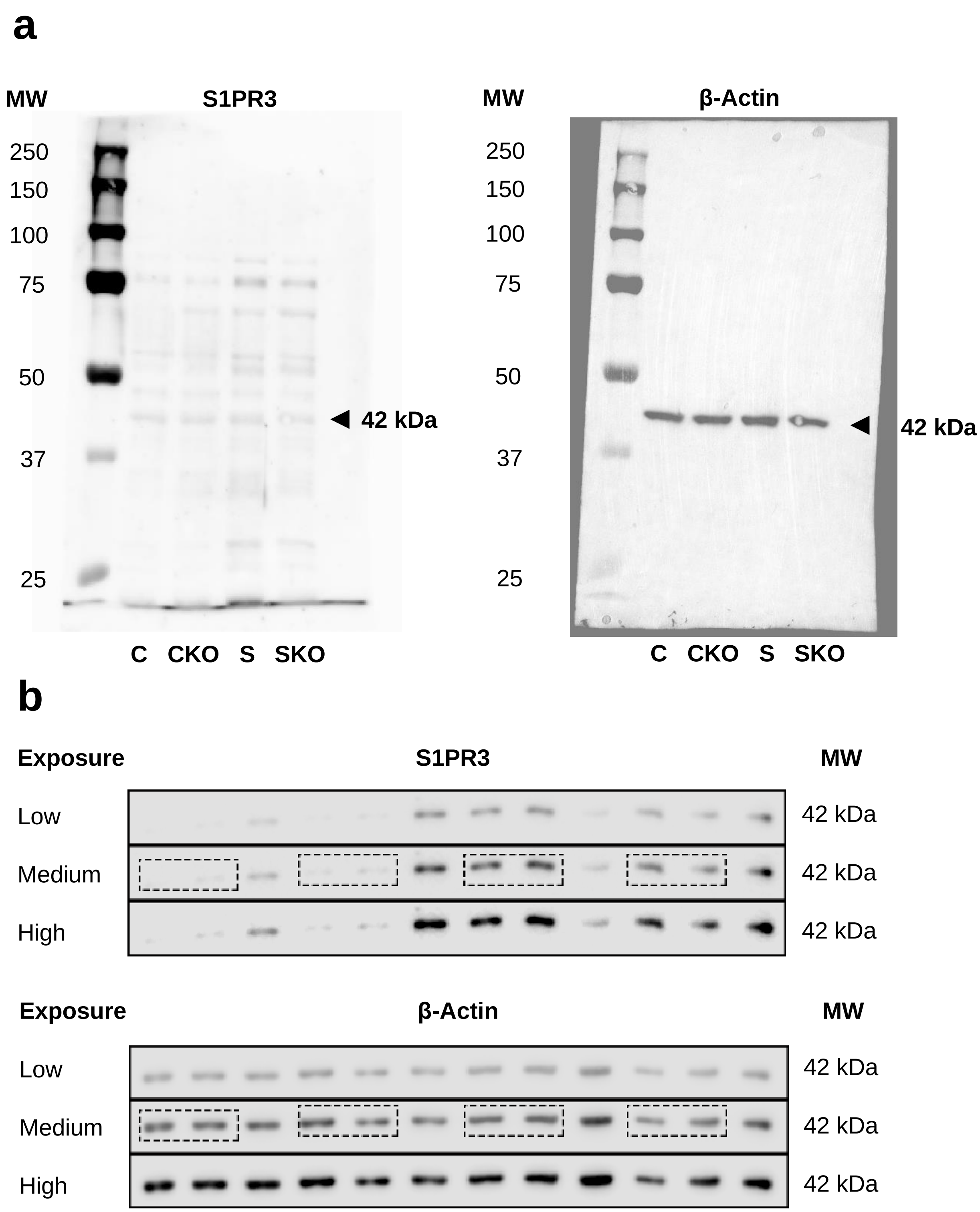

Supplementary Fig.S4 Western blot images of S1PR3. a) Full-length representative immunoblotting showing the S1PR3 band (C=Control WT; CKO=Control Sphk2-/; S=Stress WT; SKO= Stress Sphk2-/-). b) Multiple exposures (low, medium, high) of S1PR3 blots. The membranes were cut prior to hybridization. Dashed line boxes indicate the cropped images used in Fig.1l

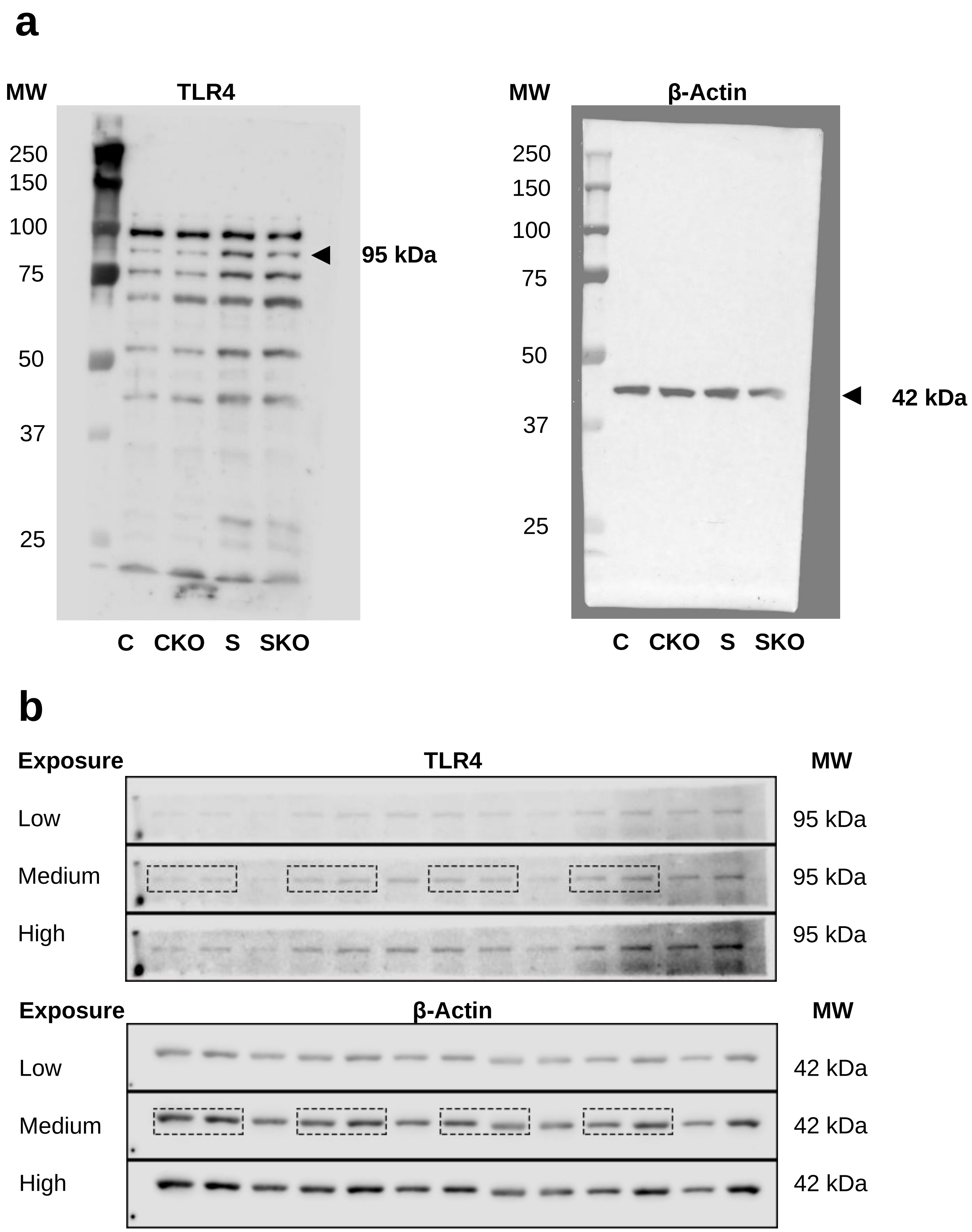

Supplementary Fig.S5 Western blot images of TLR4. a) Full-length representative immunoblotting showing the TLR4 band (C=Control WT; CKO=Control Sphk2-/; S=Stress WT; SKO= Stress Sphk2-/-). b) Multiple exposures (low, medium, high) of TLR4 blots. The membranes were cut prior to hybridization. Dashed line boxes indicate the cropped images used in Fig.2d

a

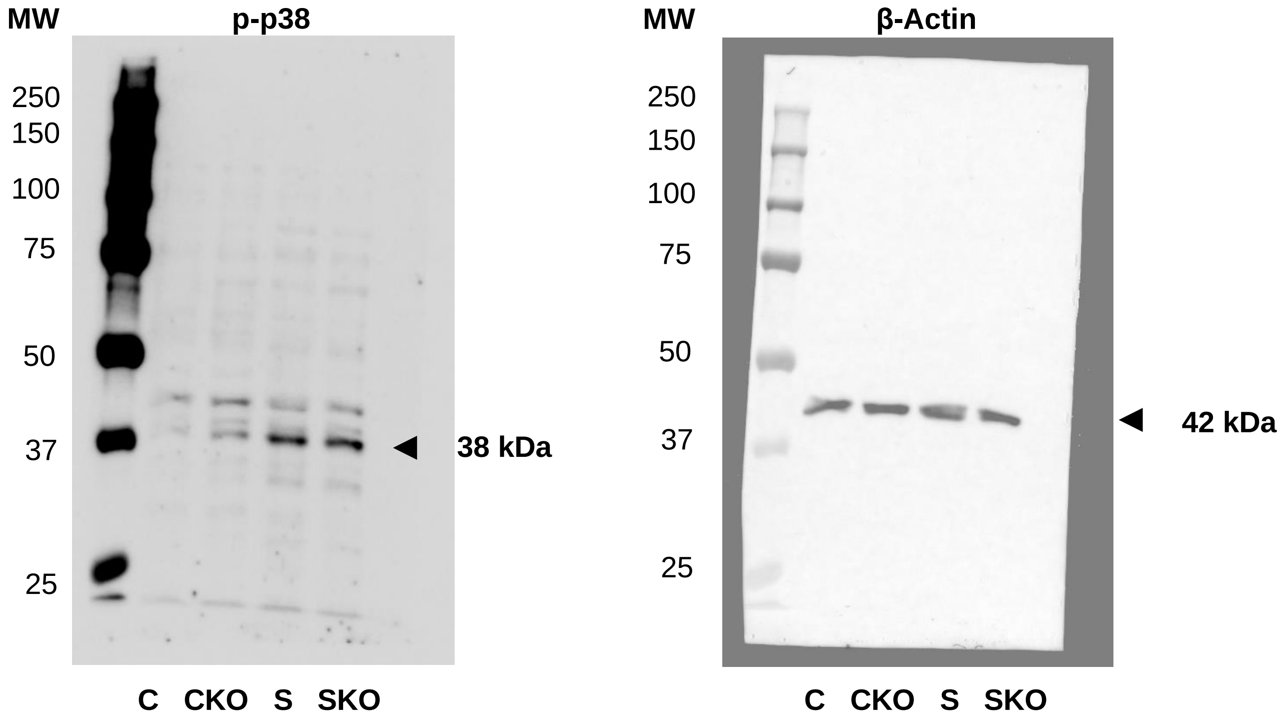

b

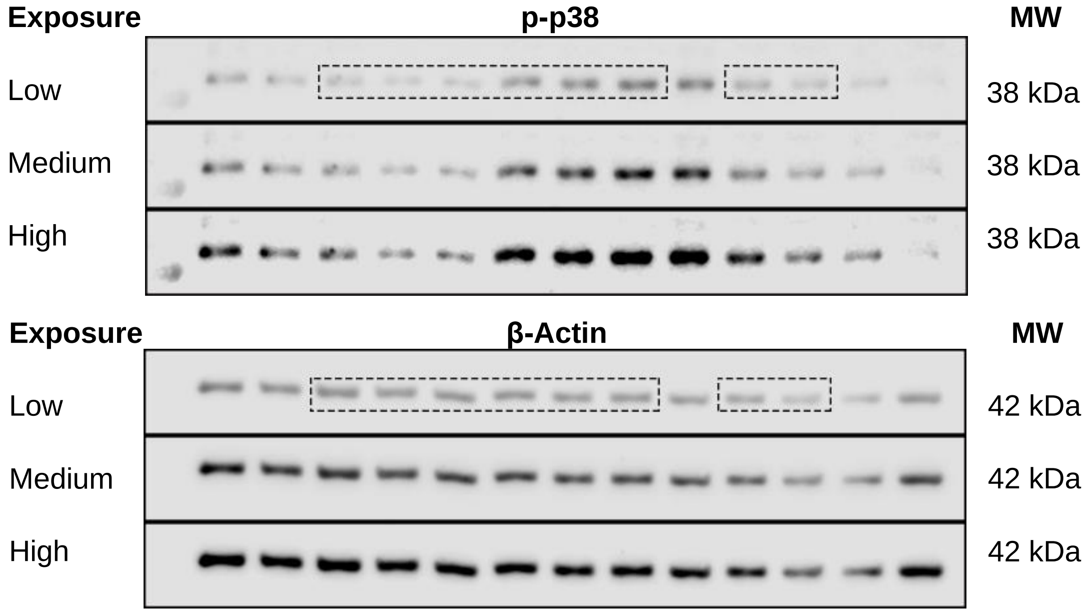

Supplementary Fig.S6 Western blot images of p-p38. a) Full-length representative immunoblotting showing the p-p38 band (C=Control WT; CKO=Control Sphk2-/; S=Stress WT; SKO= Stress Sphk2-/-). b) Multiple exposures (low, medium, high) of p-p38 blots. The membranes were cut prior to hybridization. Dashed line boxes indicate the cropped images used in Fig.2e

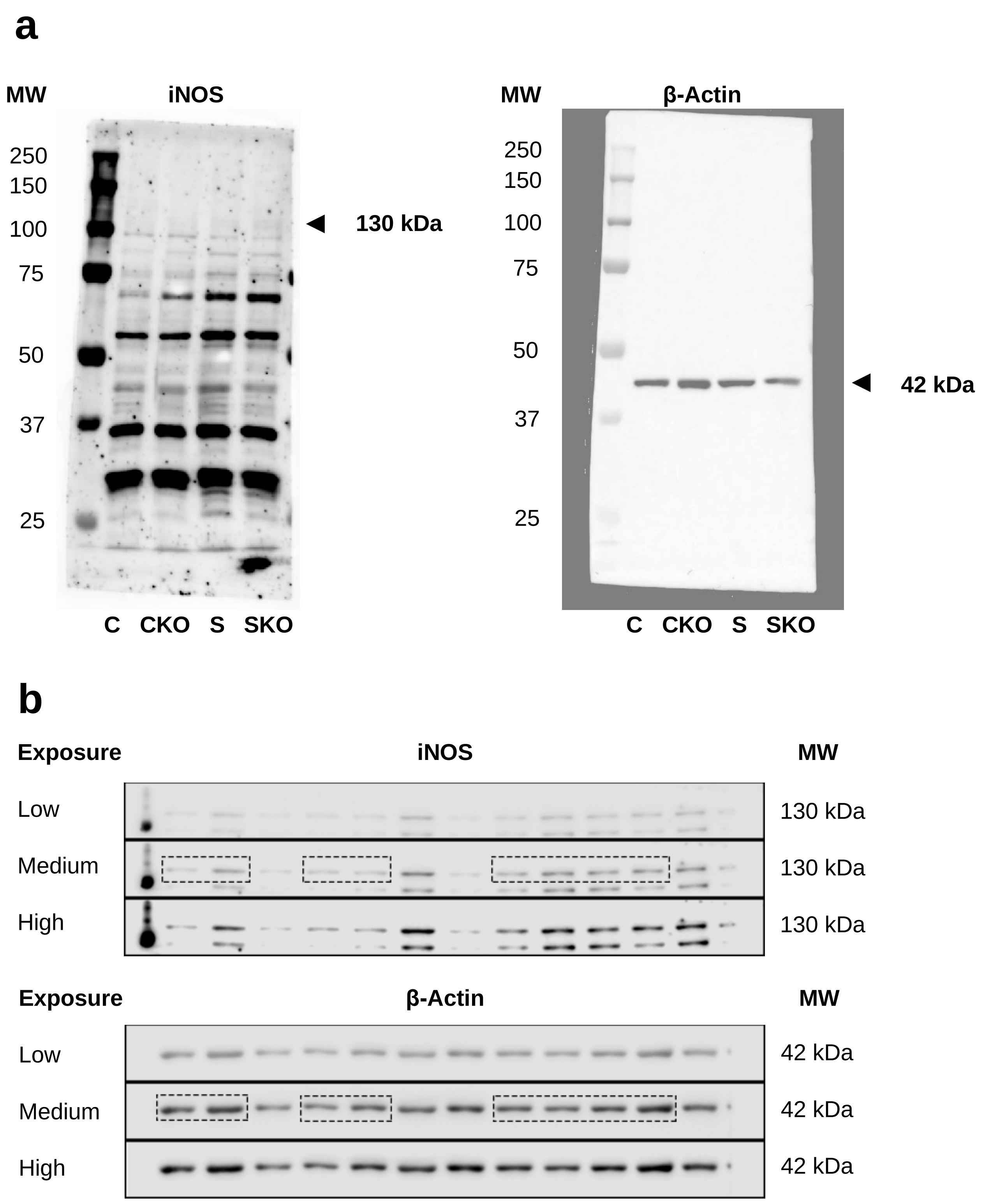

Supplementary Fig.S7 Western blot images of iNOS. a) Full-length representative immunoblotting showing the iNOS band (C=Control WT; CKO=Control Sphk2-/; S=Stress WT; SKO= Stress Sphk2-/-). b) Multiple exposures (low, medium, high) of p-iNOS blots. The membranes were cut prior to hybridization. Dashed line boxes indicate the cropped images used in Fig.2f

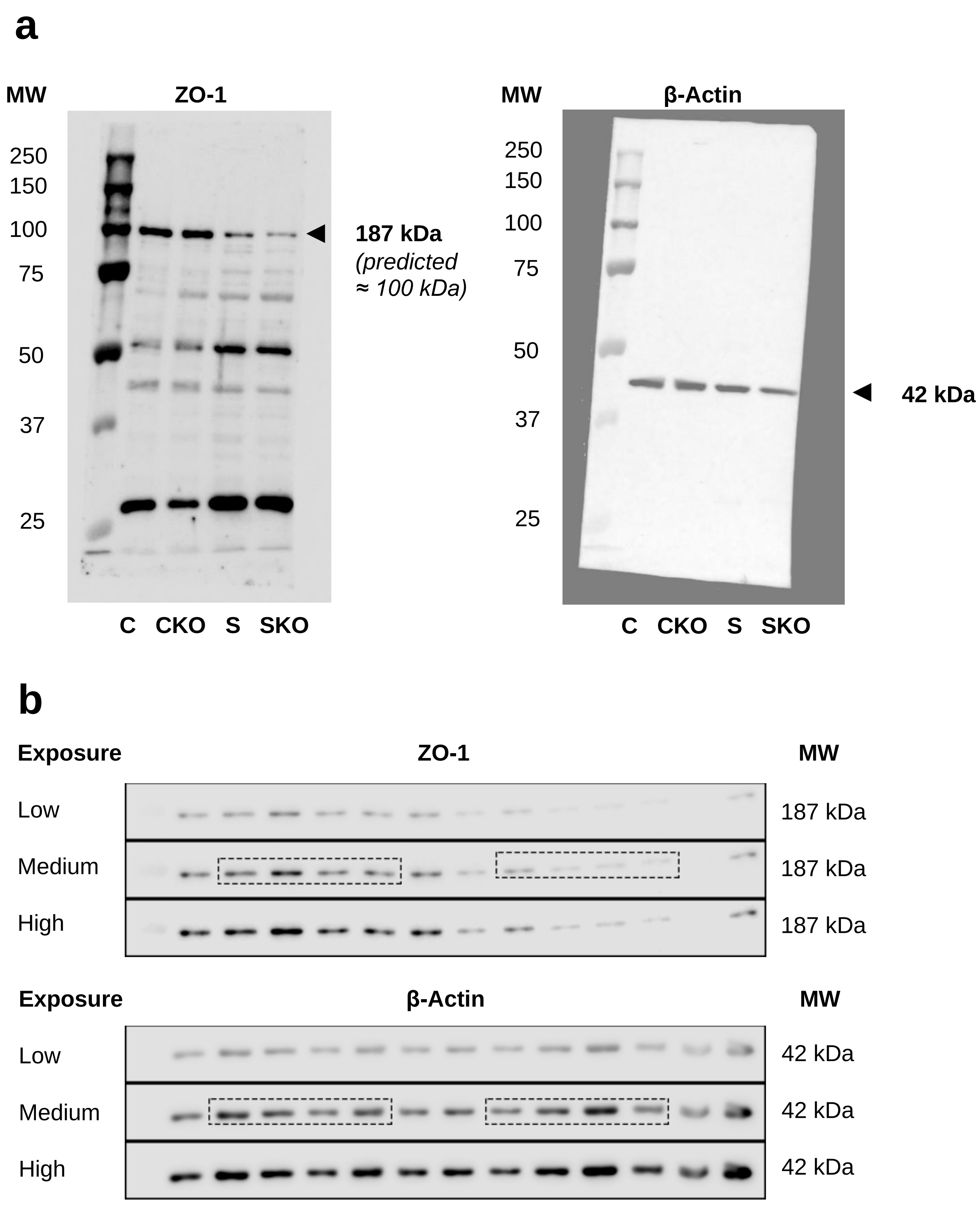

Supplementary Fig.S8 Western blot images of ZO-1. a) Full-length representative immunoblotting showing the ZO-1 band (C=Control WT; CKO=Control Sphk2-/; S=Stress WT; SKO= Stress Sphk2-/-). b) Multiple exposures (low, medium, high) of p-ZO-1 blots. The membranes were cut prior to hybridization. Dashed line boxes indicate the cropped images used in Fig.6d

Supplementary Methods: Statistical analyses - correlation analyses

Pearson's correlations between several parameters were made to further support manuscript discussion. A p value <0.05 was considered statistically significant. To account for multiple testing in the correlations, we adjusted the p-values by controlling the false discovery rate (FDR). The original FDR method of Benjamini and Hochberg with a desired FDR (q) of 0.1 was performed to restrict the occurrence of false-positive findings (type I error) among all nominal significant findings. Data were analyzed using GraphPad Prism 8 (GraphPad Software, San Diego, CA, USA).

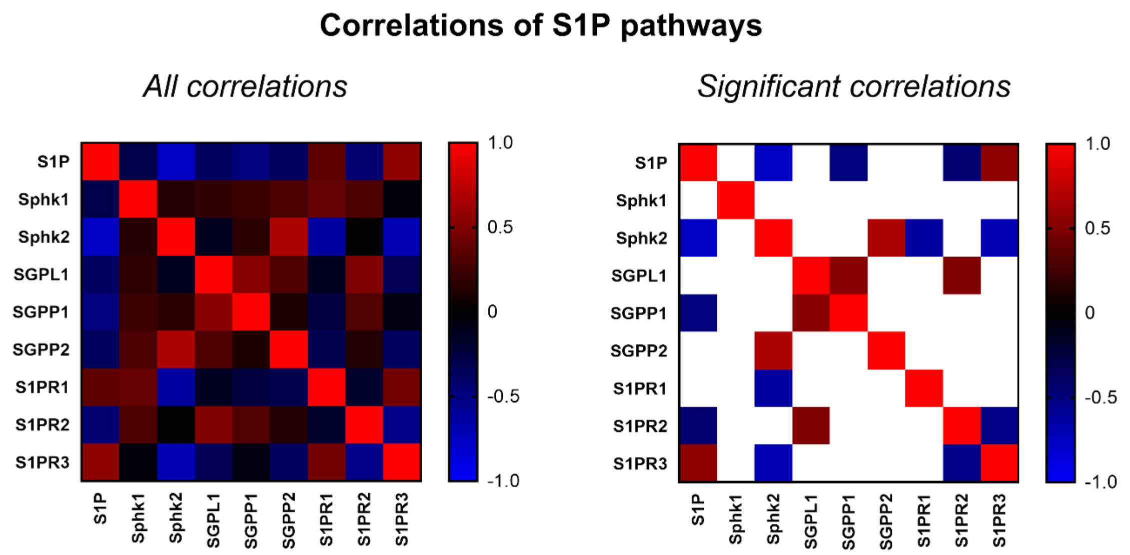

Supplementary Fig.S9 Correlations of S1P pathways. Pearson correlation coefficient (r) is represented in a double gradient from r=1 (red) to r=-1 (blue). Each square represents the interaction between two variables, one horizontal and the other one vertical. On the left, all correlations are depicted. On the right, only significant correlations (p<0.05 and q<0.1) are plotted

	S1P	Sphk1	Sphk2	SGPL1	SGPP1	SGPP2	S1PR1	S1PR2	S1PR3
**S1P**	**r=1**	r=-0.296<br>p=0.151<br>q=0.151	**r=-0.768<br>p=0.006<br>q=0.023**	r=-0.388<br>p=0.055<br>q=0.083	**r=-0.481<br>p=0.015<br>q=0.040**	r=-0.374<br>p=0.065<br>q=0.083	r=0.365<br>p=0.073<br>q=0.083	**r=-0.459<br>p=0.021<br>q=0.042**	**r=0.552<br>p=0.005<br>q=0.023**
**Sphk1**	r=-0.296<br>p=0.151<br>q=0.151	**r=1**	r=0.148<br>p=0.647<br>q=0.739	r=0.188<br>p=0.358<br>q=0.477	r=0.226<br>p=0.267<br>q=0.427	r=0.283<br>p=0.162<br>q=0.324	r=0.388<br>p=0.050<br>q=0.324	r=0.305<br>p=0.130<br>q=0.324	r=-0.047<br>p=0.822<br>q=0.822
**Sphk2**	**r=-0.768<br>p=0.006<br>q=0.023**	r=0.148<br>p=0.647<br>q=0.739	**r=1**	r=-0.124<br>p=0.702<br>q=0.802	r=0.174<br>p=0.589<br>q=0.802	**r=0.677<br>p=0.016<br>q=0.042**	**r=-0.625<br>p=0.030<br>q=0.059**	r=0.007<br>p=0.982<br>q=0.982	**r=-0.710<br>p=0.010<br>q=0.039**
**SGPL1**	r=-0.388<br>p=0.055<br>q=0.083	r=0.188<br>p=0.358<br>q=0.477	r=-0.124<br>p=0.702<br>q=0.802	**r=1**	**r=0.532<br>p=0.005<br>q=0.034**	r=0.311<br>p=0.122<br>q=0.196	r=-0.143<br>p=0.487<br>q=0.556	**r=0.505<br>p=0.008<br>q=0.034**	r=-0.338<br>p=0.098<br>q=0.196
**SGPP1**	**r=-0.481<br>p=0.015<br>q=0.040**	r=0.226<br>p=0.267<br>q=0.427	r=0.174<br>p=0.589<br>q=0.802	**r=0.532<br>p=0.005<br>q=0.034**	**r=1**	r=0.119<br>p=0.562<br>q=0.673	r=-0.244<br>p=0.230<br>q=0.427	r=0.315<br>p=0.117<br>q=0.311	r=-0.068<br>p=0.748<br>q=0.748
**SGPP2**	r=-0.374<br>p=0.065<br>q=0.083	r=0.283<br>p=0.162<br>q=0.324	**r=0.677<br>p=0.016<br>q=0.042**	r=0.311<br>p=0.122<br>q=0.196	r=0.119<br>p=0.562<br>q=0.673	**r=1**	r=-0.317<br>p=0.114<br>q=0.196	r=0.124<br>p=0.548<br>q=0.562	r=-0.384<br>p=0.058<br>q=0.174
**S1PR1**	r=0.365<br>p=0.073<br>q=0.083	r=0.388<br>p=0.050<br>q=0.324	**r=-0.625<br>p=0.030<br>q=0.059**	r=-0.143<br>p=0.487<br>q=0.556	r=-0.244<br>p=0.230<br>q=0.427	r=-0.317<br>p=0.114<br>q=0.196	**r=1**	r=-0.187<br>p=0.360<br>q=0.411	r=0.457<br>p=0.022<br>q=0.119
**S1PR2**	**r=-0.459<br>p=0.021<br>q=0.042**	r=0.305<br>p=0.130<br>q=0.324	r=0.007<br>p=0.982<br>q=0.982	**r=0.505<br>p=0.008<br>q=0.034**	r=0.315<br>p=0.117<br>q=0.311	r=0.124<br>p=0.548<br>q=0.562	r=-0.187<br>p=0.360<br>q=0.411	**r=1**	**r=-0.531<br>p=0.006<br>q=0.034**
**S1PR3**	**r=0.552<br>p=0.005<br>q=0.023**	r=-0.047<br>p=0.822<br>q=0.822	**r=-0.710<br>p=0.010<br>q=0.039**	r=-0.338<br>p=0.098<br>q=0.196	r=-0.068<br>p=0.748<br>q=0.748	r=-0.384<br>p=0.058<br>q=0.174	r=0.457<br>p=0.022<br>q=0.119	**r=-0.531<br>p=0.006<br>q=0.034**	**r=1**

Supplementary Table S2 Correlations of S1P pathways. Pearson correlation coefficient (r), p-value (p), and False Discovery Rate (q). Each square represents the interaction between two variables, one horizontal and the other one vertical. Significant correlations (p<0.05 and q<0.1) are shaded and highlighted in bold

Correlations of S1P/Sphk2 with inflammatory parameters

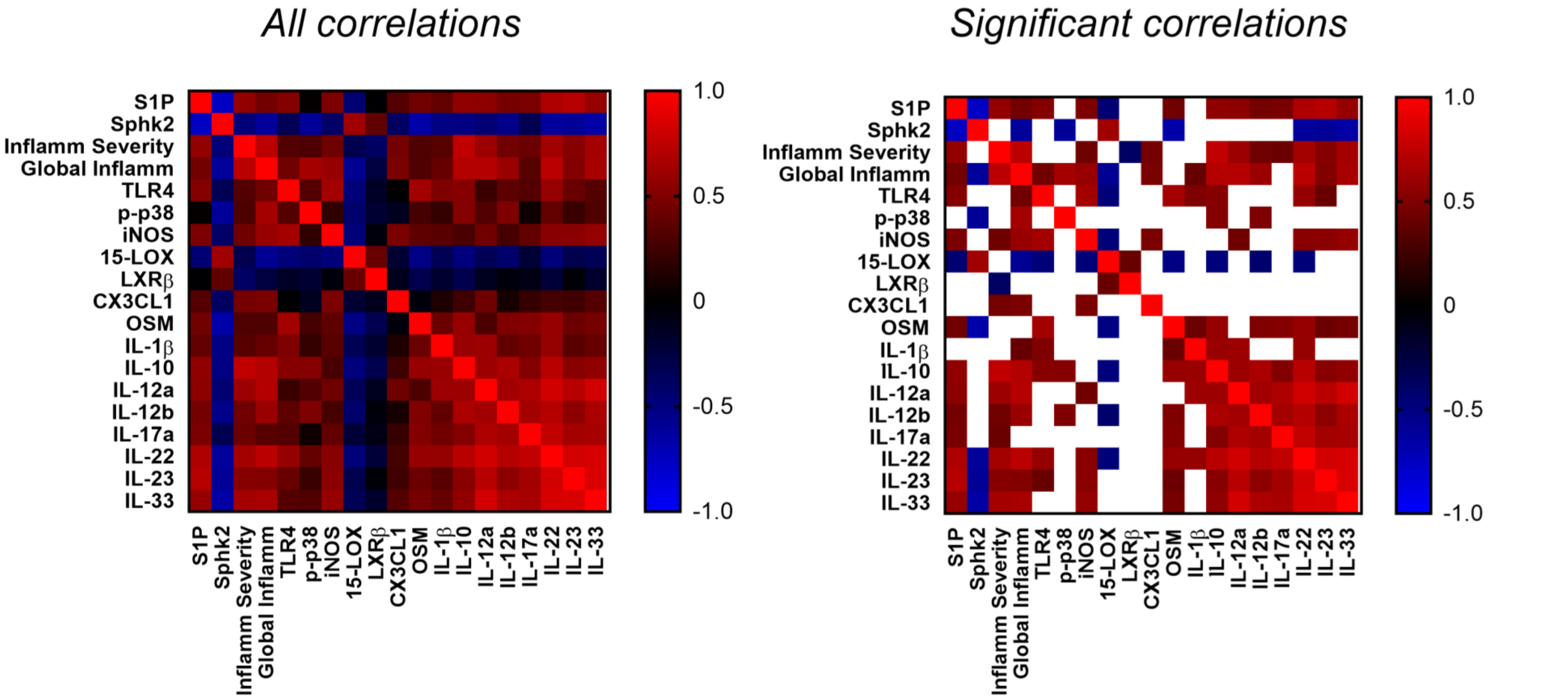

Supplementary Fig.S10 Correlations of S1P/Sphk2 with inflammatory parameters. Pearson correlation coefficient (r) is represented in a double gradient from r=1 (red) to r=-1 (blue). Each square represents the interaction between two variables, one horizontal and the other one vertical. On the left, all correlations are depicted. On the right, only significant correlations (p<0.05 and q<0.1) are plotted

	S1P	Sphk2	Inflam S	G Inflam	TLR4	p-p38	iNOS	15-LOX	LXRβ	CX3CL1	OSM	IL-1β	IL-10	IL-12a	IL-12b	IL-17a	IL-22	IL-23	IL-33
**S1P**	r=1	**r=-0.768<br>p=0.006<br>q=0.015**	**r=0.573<br>p=0.003<br>q=0.012**	**r=0.446<br>p=0.026<br>q=0.035**	**r=0.514<br>p=0.010<br>q=0.023**	r=0.016<br>p=0.938<br>q=0.942	**r=0.484<br>p=0.019<br>q=0.034**	**r=-0.455<br>p=0.026<br>q=0.035**	r=-0.015<br>p=0.942<br>q=0.942	r=0.329<br>p=0.116<br>q=0.131	**r=0.441<br>p=0.027<br>q=0.035**	r=0.383<br>p=0.065<br>q=0.078	**r=0.563<br>p=0.004<br>q=0.015**	**r=0.548<br>p=0.006<br>q=0.015**	**r=0.455<br>p=0.022<br>q=0.035**	**r=0.476<br>p=0.016<br>q=0.032**	**r=0.682<br>p<0.001<br>q=0.002**	**r=0.710<br>p<0.001<br>q=0.002**	**r=0.592<br>p=0.002<br>q=0.012**
**Sphk2**	**r=-0.768<br>p=0.006<br>q=0.015**	r=1	r=-0.472<br>p=0.121<br>q=0.182	**r=-0.605<br>p=0.037<br>q=0.094**	r=-0.334<br>p=0.289<br>q=0.306	**r=-0.601<br>p=0.039<br>q=0.094**	r=-0.402<br>p=0.220<br>q=0.256	**r=0.625<br>p=0.040<br>q=0.094**	r=0.377<br>p=0.227<br>q=0.256	r=-0.386<br>p=0.215<br>q=0.256	**r=-0.666<br>p=0.018<br>q=0.094**	r=-0.517<br>p=0.085<br>q=0.143	r=-0.514<br>p=0.088<br>q=0.143	r=-0.447<br>p=0.145<br>q=0.201	r=-0.539<br>p=0.071<br>q=0.141	r=-0.318<br>p=0.314<br>q=0.314	**r=-0.611<br>p=0.035<br>q=0.094**	**r=-0.594<br>p=0.042<br>q=0.094**	**r=-0.645<br>p=0.023<br>q=0.094**
**Inflam S**	**r=0.573<br>p=0.003<br>q=0.012**	r=-0.472<br>p=0.121<br>q=0.182	r=1	**r=0.728<br>p<0.001<br>q<0.001**	r=0.322<br>p=0.116<br>q=0.136	r=0.296<br>p=0.142<br>q=0.142	**r=0.434<br>p=0.034<br>q=0.056**	r=-0.312<br>p=0.129<br>q=0.136	**r=-0.392<br>p=0.048<br>q=0.072**	**r=0.451<br>p=0.024<br>q=0.048**	r=0.308<br>p=0.126<br>q=0.136	r=0.334<br>p=0.103<br>q=0.136	**r=0.750<br>p<0.001<br>q<0.001**	**r=0.610<br>p=0.001<br>q=0.004**	**r=0.442<br>p=0.024<br>q=0.048**	**r=0.419<br>p=0.033<br>q=0.056**	**r=0.636<br>p=0.001<br>q=0.003**	**r=0.522<br>p=0.006<br>q=0.016**	**r=0.641<br>p=0.001<br>q=0.003**
**G Inflam**	**r=0.446<br>p=0.026<br>q=0.035**	**r=-0.605<br>p=0.037<br>q=0.094**	**r=0.728<br>p<0.001<br>q<0.001**	r=1	**r=0.440<br>p=0.028<br>q=0.038**	**r=0.640<br>p<0.001<br>q=0.001**	**r=0.595<br>p=0.002<br>q=0.005**	**r=-0.577<br>p=0.003<br>q=0.005**	r=-0.247<br>p=0.224<br>q=0.224	**r=0.479<br>p=0.015<br>q=0.025**	r=0.310<br>p=0.124<br>q=0.131	**r=0.400<br>p=0.048<br>q=0.057**	**r=0.700<br>p<0.001<br>q<0.001**	**r=0.704<br>p<0.001<br>q<0.001**	**r=0.606<br>p=0.001<br>q=0.003**	r=0.338<br>p=0.091<br>q=0.102	**r=0.730<br>p<0.001<br>q<0.001**	**r=0.510<br>p=0.008<br>q=0.014**	**r=0.659<br>p<0.001<br>q=0.001**
**TLR4**	**r=0.514<br>p=0.010<br>q=0.023**	r=-0.334<br>p=0.289<br>q=0.306	r=0.322<br>p=0.116<br>q=0.136	**r=0.440<br>p=0.028<br>q=0.038**	r=1	r=0.329<br>p=0.108<br>q=0.152	**r=0.641<br>p=0.001<br>q=0.009**	**r=-0.476<br>p=0.019<br>q=0.048**	r=-0.151<br>p=0.470<br>q=0.498	r=-0.002<br>p=0.992<br>q=0.992	**r=0.638<br>p=0.001<br>q=0.009**	**r=0.496<br>p=0.014<br>q=0.041**	**r=0.528<br>p=0.008<br>q=0.036**	r=0.224<br>p=0.293<br>q=0.329	r=0.360<br>p=0.077<br>q=0.139	r=0.333<br>p=0.104<br>q=0.152	**r=0.584<br>p=0.003<br>q=0.016**	**r=0.409<br>p=0.042<br>q=0.085**	r=0.328<br>p=0.118<br>q=0.152
**p-p38**	r=0.016<br>p=0.938<br>q=0.942	**r=-0.601<br>p=0.039<br>q=0.094**	r=0.296<br>p=0.142<br>q=0.142	**r=0.640<br>p<0.001<br>q=0.001**	r=0.329<br>p=0.108<br>q=0.152	r=1	r=0.185<br>p=0.386<br>q=0.464	r=-0.427<br>p=0.033<br>q=0.140	r=-0.198<br>p=0.333<br>q=0.428	r=-0.122<br>p=0.562<br>q=0.632	r=0.271<br>p=0.181<br>q=0.297	r=0.203<br>p=0.330<br>q=0.428	**r=0.519<br>p=0.008<br>q=0.058**	r=0.318<br>p=0.121<br>q=0.249	**r=0.497<br>p=0.010<br>q=0.058**	r=0.055<br>p=0.791<br>q=0.838	r=0.382<br>p=0.059<br>q=0.178	r=0.222<br>p=0.276<br>q=0.415	r=0.315<br>p=0.125<br>q=0.249
**iNOS**	**r=0.484<br>p=0.019<br>q=0.034**	r=-0.402<br>p=0.220<br>q=0.256	**r=0.434<br>p=0.034<br>q=0.056**	**r=0.595<br>p=0.002<br>q=0.005**	**r=0.641<br>p=0.001<br>q=0.009**	r=0.185<br>p=0.386<br>q=0.464	r=1	**r=-0.477<br>p=0.021<br>q=0.048**	r=-0.053<br>p=0.807<br>q=0.807	**r=0.479<br>p=0.021<br>q=0.048**	r=0.366<br>p=0.079<br>q=0.118	r=0.345<br>p=0.107<br>q=0.148	r=0.279<br>p=0.197<br>q=0.236	**r=0.437<br>p=0.037<br>q=0.067**	r=0.277<br>p=0.190<br>q=0.236	r=0.382<br>p=0.065<br>q=0.107	**r=0.532<br>p=0.009<br>q=0.032**	**r=0.540<br>p=0.006<br>q=0.029**	**r=0.584<br>p=0.003<br>q=0.021**
**15-LOX**	**r=-0.455<br>p=0.026<br>q=0.035**	**r=0.625<br>p=0.040<br>q=0.094**	r=-0.312<br>p=0.129<br>q=0.136	**r=-0.577<br>p=0.003<br>q=0.005**	**r=-0.476<br>p=0.019<br>q=0.048**	r=-0.427<br>p=0.033<br>q=0.140	**r=-0.477<br>p=0.021<br>q=0.048**	r=1	**r=0.410<br>p=0.042<br>q=0.069**	r=-0.201<br>p=0.346<br>q=0.346	**r=-0.524<br>p=0.007<br>q=0.064**	r=-0.335<br>p=0.110<br>q=0.141	**r=-0.489<br>p=0.015<br>q=0.064**	r=-0.340<br>p=0.104<br>q=0.141	**r=-0.424<br>p=0.035<br>q=0.069**	r=-0.215<br>p=0.303<br>q=0.321	**r=-0.473<br>p=0.020<br>q=0.064**	r=-0.302<br>p=0.142<br>q=0.160	r=-0.335<br>p=0.110<br>q=0.141
**LXRβ**	r=-0.015<br>p=0.942<br>q=0.942	r=0.377<br>p=0.227<br>q=0.256	**r=-0.392<br>p=0.048<br>q=0.072**	r=-0.247<br>p=0.224<br>q=0.224	r=-0.151<br>p=0.470<br>q=0.498	r=-0.198<br>p=0.333<br>q=0.428	r=-0.053<br>p=0.807<br>q=0.807	**r=0.410<br>p=0.042<br>q=0.069**	r=1	r=-0.114<br>p=0.587<br>q=0.813	r=-0.306<br>p=0.128<br>q=0.637	r=-0.190<br>p=0.362<br>q=0.712	r=-0.302<br>p=0.142<br>q=0.637	r=-0.125<br>p=0.552<br>q=0.813	r=-0.044<br>p=0.831<br>q=0.935	r=-0.084<br>p=0.682<br>q=0.876	r=-0.227<br>p=0.275<br>q=0.706	r=-0.030<br>p=0.884<br>q=0.936	r=-0.178<br>p=0.395<br>q=0.712
**CX3CL1**	r=0.329<br>p=0.116<br>q=0.131	r=-0.386<br>p=0.215<br>q=0.256	**r=0.451<br>p=0.024<br>q=0.048**	**r=0.479<br>p=0.015<br>q=0.025**	r=-0.002<br>p=0.992<br>q=0.992	r=-0.122<br>p=0.562<br>q=0.632	**r=0.479<br>p=0.021<br>q=0.048**	r=-0.201<br>p=0.346<br>q=0.346	r=-0.114<br>p=0.587<br>q=0.813	r=1	r=-0.053<br>p=0.800<br>q=0.847	r=0.117<br>p=0.587<br>q=0.705	r=0.241<br>p=0.245<br>q=0.441	r=0.432<br>p=0.031<br>q=0.139	r=0.090<br>p=0.669<br>q=0.752	r=0.213<br>p=0.307<br>q=0.503	r=0.270<br>p=0.193<br>q=0.441	r=0.248<br>p=0.232<br>q=0.441	r=0.326<br>p=0.112<br>q=0.348
**OSM**	**r=0.441<br>p=0.027<br>q=0.035**	**r=-0.666<br>p=0.018<br>q=0.094**	r=0.308<br>p=0.126<br>q=0.136	r=0.310<br>p=0.124<br>q=0.131	**r=0.638<br>p=0.001<br>q=0.009**	r=0.271<br>p=0.181<br>q=0.297	r=0.366<br>p=0.079<br>q=0.118	**r=-0.524<br>p=0.007<br>q=0.064**	r=-0.306<br>p=0.128<br>q=0.637	r=-0.053<br>p=0.800<br>q=0.847	r=1	**r=0.414<br>p=0.039<br>q=0.065**	**r=0.588<br>p=0.002<br>q=0.012**	r=0.296<br>p=0.150<br>q=0.169	**r=0.500<br>p=0.009<br>q=0.028**	**r=0.514<br>p=0.007<br>q=0.026**	**r=0.586<br>p=0.002<br>q=0.012**	**r=0.423<br>p=0.031<br>q=0.056**	**r=0.462<br>p=0.020<br>q=0.045**
**IL-1β**	r=0.383<br>p=0.065<br>q=0.078	r=-0.517<br>p=0.085<br>q=0.143	r=0.334<br>p=0.103<br>q=0.136	**r=0.400<br>p=0.048<br>q=0.057**	**r=0.496<br>p=0.014<br>q=0.041**	r=0.203<br>p=0.330<br>q=0.428	r=0.345<br>p=0.107<br>q=0.148	r=-0.335<br>p=0.110<br>q=0.141	r=-0.190<br>p=0.362<br>q=0.712	r=0.117<br>p=0.587<br>q=0.705	**r=0.414<br>p=0.039<br>q=0.065**	r=1	**r=0.599<br>p=0.002<br>q=0.018**	**r=0.610<br>p=0.002<br>q=0.018**	r=0.381<br>p=0.060<br>q=0.117	r=0.435<br>p=0.030<br>q=0.107	**r=0.581<br>p=0.003<br>q=0.018**	r=0.342<br>p=0.094<br>q=0.132	r=0.399<br>p=0.053<br>q=0.117
**IL-10**	**r=0.563<br>p=0.004<br>q=0.015**	r=-0.514<br>p=0.088<br>q=0.143	**r=0.750<br>p<0.001<br>q<0.001**	**r=0.700<br>p<0.001<br>q<0.001**	**r=0.528<br>p=0.008<br>q=0.036**	**r=0.519<br>p=0.008<br>q=0.058**	r=0.279<br>p=0.197<br>q=0.236	**r=-0.489<br>p=0.015<br>q=0.064**	r=-0.302<br>p=0.142<br>q=0.637	r=0.241<br>p=0.245<br>q=0.441	**r=0.588<br>p=0.002<br>q=0.012**	**r=0.599<br>p=0.002<br>q=0.018**	r=1	**r=0.610<br>p=0.001<br>q=0.004**	**r=0.667<br>p<0.001<br>q=0.001**	**r=0.512<br>p=0.009<br>q=0.012**	**r=0.723<br>p<0.001<br>q<0.001**	**r=0.533<br>p=0.006<br>q=0.011**	**r=0.566<br>p=0.003<br>q=0.007**
**IL-12a**	**r=0.548<br>p=0.006<br>q=0.015**	r=-0.447<br>p=0.145<br>q=0.201	**r=0.610<br>p=0.001<br>q=0.004**	**r=0.704<br>p<0.001<br>q<0.001**	r=0.224<br>p=0.293<br>q=0.329	r=0.318<br>p=0.121<br>q=0.249	**r=0.437<br>p=0.037<br>q=0.067**	r=-0.340<br>p=0.104<br>q=0.141	r=-0.125<br>p=0.552<br>q=0.813	r=0.432<br>p=0.031<br>q=0.139	r=0.296<br>p=0.150<br>q=0.169	**r=0.610<br>p=0.002<br>q=0.018**	**r=0.610<br>p=0.001<br>q=0.004**	r=1	**r=0.634<br>p=0.001<br>q=0.002**	**r=0.676<br>p<0.001<br>q=0.001**	**r=0.817<br>p<0.001<br>q<0.001**	**r=0.719<br>p<0.001<br>q<0.001**	**r=0.824<br>p<0.001<br>q<0.001**
**IL-12b**	**r=0.455<br>p=0.022<br>q=0.035**	r=-0.539<br>p=0.071<br>q=0.141	**r=0.442<br>p=0.024<br>q=0.048**	**r=0.606<br>p=0.001<br>q=0.003**	r=0.360<br>p=0.077<br>q=0.139	**r=0.497<br>p=0.010<br>q=0.058**	r=0.277<br>p=0.190<br>q=0.236	**r=-0.424<br>p=0.035<br>q=0.069**	r=-0.044<br>p=0.831<br>q=0.935	r=0.090<br>p=0.669<br>q=0.752	**r=0.500<br>p=0.009<br>q=0.028**	r=0.381<br>p=0.060<br>q=0.117	**r=0.667<br>p<0.001<br>q=0.001**	**r=0.634<br>p=0.001<br>q=0.002**	r=1	**r=0.646<br>p<0.001<br>q=0.002**	**r=0.708<br>p<0.001<br>q=0.001**	**r=0.548<br>p=0.004<br>q=0.010**	**r=0.669<br>p<0.001<br>q=0.002**
**IL-17a**	**r=0.476<br>p=0.016<br>q=0.032**	r=-0.318<br>p=0.314<br>q=0.314	**r=0.419<br>p=0.033<br>q=0.056**	r=0.338<br>p=0.091<br>q=0.102	r=0.333<br>p=0.104<br>q=0.152	r=0.055<br>p=0.791<br>q=0.838	r=0.382<br>p=0.065<br>q=0.107	r=-0.215<br>p=0.303<br>q=0.321	r=-0.084<br>p=0.682<br>q=0.876	r=0.213<br>p=0.307<br>q=0.503	**r=0.514<br>p=0.007<br>q=0.026**	r=0.435<br>p=0.030<br>q=0.107	**r=0.512<br>p=0.009<br>q=0.012**	**r=0.676<br>p<0.001<br>q=0.001**	**r=0.646<br>p<0.001<br>q=0.002**	r=1	**r=0.755<br>p<0.001<br>q<0.001**	**r=0.638<br>p<0.001<br>q=0.002**	**r=0.654<br>p<0.001<br>q=0.002**
**IL-22**	**r=0.682<br>p<0.001<br>q=0.002**	**r=-0.611<br>p=0.035<br>q=0.094**	**r=0.636<br>p=0.001<br>q=0.003**	**r=0.730<br>p<0.001<br>q<0.001**	**r=0.584<br>p=0.003<br>q=0.016**	r=0.382<br>p=0.059<br>q=0.178	**r=0.532<br>p=0.009<br>q=0.032**	**r=-0.473<br>p=0.020<br>q=0.064**	r=-0.227<br>p=0.275<br>q=0.706	r=0.270<br>p=0.193<br>q=0.441	**r=0.586<br>p=0.002<br>q=0.012**	**r=0.581<br>p=0.003<br>q=0.018**	**r=0.723<br>p<0.001<br>q<0.001**	**r=0.817<br>p<0.001<br>q<0.001**	**r=0.708<br>p<0.001<br>q=0.001**	**r=0.755<br>p<0.001<br>q<0.001**	r=1	**r=0.827<br>p<0.001<br>q<0.001**	**r=0.839<br>p<0.001<br>q<0.001**
**IL-23**	**r=0.710<br>p<0.001<br>q=0.002**	**r=-0.594<br>p=0.042<br>q=0.094**	**r=0.522<br>p=0.006<br>q=0.016**	**r=0.510<br>p=0.008<br>q=0.014**	**r=0.409<br>p=0.042<br>q=0.085**	r=0.222<br>p=0.276<br>q=0.415	**r=0.540<br>p=0.006<br>q=0.029**	r=-0.302<br>p=0.142<br>q=0.160	r=-0.030<br>p=0.884<br>q=0.936	r=0.248<br>p=0.232<br>q=0.441	**r=0.423<br>p=0.031<br>q=0.056**	r=0.342<br>p=0.094<br>q=0.132	**r=0.533<br>p=0.006<br>q=0.011**	**r=0.719<br>p<0.001<br>q<0.001**	**r=0.548<br>p=0.004<br>q=0.010**	**r=0.638<br>p<0.001<br>q=0.002**	**r=0.827<br>p<0.001<br>q<0.001**	r=1	**r=0.849<br>p<0.001<br>q<0.001**
**IL-33**	**r=0.592<br>p=0.002<br>q=0.012**	**r=-0.645<br>p=0.023<br>q=0.094**	**r=0.641<br>p=0.001<br>q=0.003**	**r=0.659<br>p<0.001<br>q=0.001**	r=0.328<br>p=0.118<br>q=0.152	r=0.315<br>p=0.125<br>q=0.249	**r=0.584<br>p=0.003<br>q=0.021**	r=-0.335<br>p=0.110<br>q=0.141	r=-0.178<br>p=0.395<br>q=0.712	r=0.326<br>p=0.112<br>q=0.348	**r=0.462<br>p=0.020<br>q=0.045**	r=0.399<br>p=0.053<br>q=0.117	**r=0.566<br>p=0.003<br>q=0.007**	**r=0.824<br>p<0.001<br>q<0.001**	**r=0.669<br>p<0.001<br>q=0.002**	**r=0.654<br>p<0.001<br>q=0.002**	**r=0.839<br>p<0.001<br>q<0.001**	**r=0.849<br>p<0.001<br>q<0.001**	r=1

Supplementary Table S3 Correlations of S1P/Sphk2 with structural proteins and crypt architecture abnormalities. Pearson correlation coefficient (r), p-value (p), and False Discovery Rate (q). Each square represents the interaction between two variables, one horizontal and the other one vertical. Significant correlations (p<0.05 and q<0.1) are shaded and highlighted in bold

Correlations of S1P/Sphk2 with structural proteins and crypt architecture abnormalities

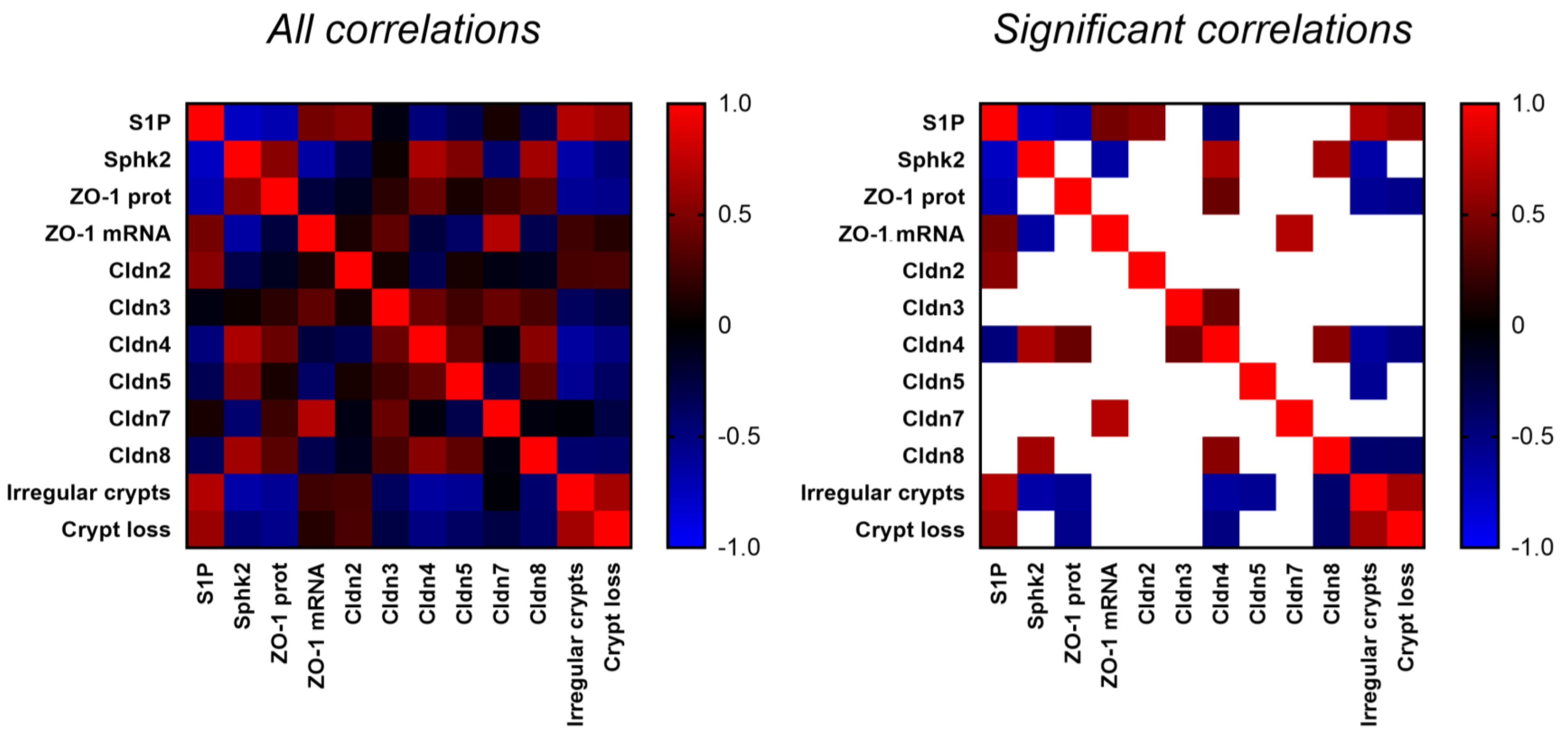

Supplementary Fig.S11 Correlations of S1P/Sphk2 with structural proteins and crypt architecture abnormalities. Pearson correlation coefficient (r) is represented in a double gradient from r=1 (red) to r=-1 (blue). Each square represents the interaction between two variables, one horizontal and the other one vertical. On the left, all correlations are depicted. On the right, only significant correlations ($p<0.05$ and $q<0.1$) are plotted

	S1P	Sphk2	ZO-1 prot	ZO-1 mRNA	Cldn2	Cldn3	Cldn4	Cldn5	Cldn7	Cldn8	Irregular crypts	Crypt loss
**S1P**	**r=1**	**r=-0,768<br>p=0.006<br>q=0.016**	**r=-0,697<br>p<0.001<br>q=0.001**	**r=0,458<br>p=0.021<br>q=0.034**	**r=0,533<br>p=0.007<br>q=0.016**	r=-0,070<br>p=0.740<br>q=0.740	**r=-0,479<br>p=0.016<br>q=0.028**	r=-0,327<br>p=0.119<br>q=0.145	r=0,096<br>p=0.648<br>q=0.713	r=-0,354<br>p=0.082<br>q=0.113	**r=0,695<br>p<0.001<br>q=0.001**	**r=0,597<br>p=0.002<br>q=0.006**
**Sphk2**	**r=-0,768<br>p=0.006<br>q=0.016**	**r=1**	r=0,533<br>p=0.074<br>q=0.136	**r=-0,637<br>p=0.026<br>q=0.059**	r=-0,292<br>p=0.383<br>q=0.421	r=0,044<br>p=0.893<br>q=0.893	**r=0,664<br>p=0.019<br>q=0.059**	r=0,491<br>p=0.105<br>q=0.165	r=-0,438<br>p=0.155<br>q=0.189	**r=0,635<br>p=0.027<br>q=0.059**	**r=-0,658<br>p=0.020<br>q=0.059**	r=-0,463<br>p=0.129<br>q=0.178
**ZO-1 prot**	**r=-0,697<br>p<0.001<br>q=0.001**	r=0,533<br>p=0.074<br>q=0.136	**r=1**	r=-0,248<br>p=0.233<br>q=0.358	r=-0,124<br>p=0.563<br>q=0.619	r=0,164<br>p=0.433<br>q=0.529	**r=0,410<br>p=0.042<br>q=0.115**	r=0,092<br>p=0.670<br>q=0.670	r=0,234<br>p=0.260<br>q=0.358	r=0,347<br>p=0.089<br>q=0.164	**r=-0,584<br>p=0.002<br>q=0.012**	**r=-0,543<br>p=0.005<br>q=0.018**
**ZO-1 mRNA**	**r=0,458<br>p=0.021<br>q=0.034**	**r=-0,637<br>p=0.026<br>q=0.059**	r=-0,248<br>p=0.233<br>q=0.358	**r=1**	r=0,102<br>p=0.629<br>q=0.629	r=0,371<br>p=0.062<br>q=0.136	r=-0,250<br>p=0.219<br>q=0.287	r=-0,399<br>p=0.048<br>q=0.132	**r=0,700<br>p<0.001<br>q=0.001**	r=-0,312<br>p=0.120<br>q=0.221	r=0,242<br>p=0.235<br>q=0.287	r=0,144<br>p=0.482<br>q=0.530
**Cldn2**	**r=0,533<br>p=0.007<br>q=0.016**	r=-0,292<br>p=0.383<br>q=0.421	r=-0,124<br>p=0.563<br>q=0.619	r=0,102<br>p=0.629<br>q=0.629	**r=1**	r=0,073<br>p=0.729<br>q=0.735	r=-0,318<br>p=0.122<br>q=0.501	r=0,084<br>p=0.696<br>q=0.735	r=-0,071<br>p=0.735<br>q=0.735	r=-0,116<br>p=0.581<br>q=0.735	r=0,276<br>p=0.182<br>q=0.501	r=0,287<br>p=0.164<br>q=0.501
**Cldn3**	r=-0,070<br>p=0.740<br>q=0.740	r=0,044<br>p=0.893<br>q=0.893	r=0,164<br>p=0.433<br>q=0.529	r=0,371<br>p=0.062<br>q=0.136	r=0,073<br>p=0.729<br>q=0.735	**r=1**	**r=0,414<br>p=0.035<br>q=0.184**	r=0,247<br>p=0.234<br>q=0.367	r=0,406<br>p=0.040<br>q=0.184	r=0,279<br>p=0.167<br>q=0.335	r=-0,365<br>p=0.067<br>q=0.184	r=-0,270<br>p=0.183<br>q=0.335
**Cldn4**	**r=-0,479<br>p=0.016<br>q=0.028**	**r=0,664<br>p=0.019<br>q=0.059**	**r=0,410<br>p=0.042<br>q=0.115**	r=-0,250<br>p=0.219<br>q=0.287	r=-0,318<br>p=0.122<br>q=0.501	**r=0,414<br>p=0.035<br>q=0.184**	**r=1**	r=0,384<br>p=0.058<br>q=0.079	r=-0,061<br>p=0.767<br>q=0.767	**r=0,535<br>p=0.005<br>q=0.027**	**r=-0,625<br>p=0.001<br>q=0.007**	**r=-0,509<br>p=0.008<br>q=0.029**
**Cldn5**	r=-0,327<br>p=0.119<br>q=0.145	r=0,491<br>p=0.105<br>q=0.165	r=0,092<br>p=0.670<br>q=0.670	r=-0,399<br>p=0.048<br>q=0.132	r=0,084<br>p=0.696<br>q=0.735	r=0,247<br>p=0.234<br>q=0.367	r=0,384<br>p=0.058<br>q=0.079	**r=1**	r=-0,296<br>p=0.150<br>q=0.207	r=0,368<br>p=0.071<br>q=0.155	**r=-0,573<br>p=0.003<br>q=0.030**	r=-0,386<br>p=0.057<br>q=0.155
**Cldn7**	r=0,096<br>p=0.648<br>q=0.713	r=-0,438<br>p=0.155<br>q=0.189	r=0,234<br>p=0.260<br>q=0.358	**r=0,700<br>p<0.001<br>q=0.001**	r=-0,071<br>p=0.735<br>q=0.735	r=0,406<br>p=0.040<br>q=0.184	r=-0,061<br>p=0.767<br>q=0.767	r=-0,296<br>p=0.150<br>q=0.207	**r=1**	r=-0,059<br>p=0.776<br>q=0.854	r=-0,032<br>p=0.878<br>q=0.878	r=-0,267<br>p=0.187<br>q=0.412
**Cldn8**	r=-0,354<br>p=0.082<br>q=0.113	**r=0,635<br>p=0.027<br>q=0.059**	r=0,347<br>p=0.089<br>q=0.164	r=-0,312<br>p=0.120<br>q=0.221	r=-0,116<br>p=0.581<br>q=0.735	r=0,279<br>p=0.167<br>q=0.335	**r=0,535<br>p=0.005<br>q=0.027**	r=0,368<br>p=0.071<br>q=0.155	r=-0,059<br>p=0.776<br>q=0.854	**r=1**	**r=-0,431<br>p=0.028<br>q=0.097**	**r=-0,414<br>p=0.035<br>q=0.097**
**Irregular crypts**	**r=0,695<br>p<0.001<br>q=0.001**	**r=-0,658<br>p=0.020<br>q=0.059**	**r=-0,584<br>p=0.002<br>q=0.012**	r=0,242<br>p=0.235<br>q=0.287	r=0,276<br>p=0.182<br>q=0.501	r=-0,365<br>p=0.067<br>q=0.184	**r=-0,625<br>p=0.001<br>q=0.007**	**r=-0,573<br>p=0.003<br>q=0.030**	r=-0,032<br>p=0.878<br>q=0.878	**r=-0,431<br>p=0.028<br>q=0.097**	**r=1**	**r=0,638<br>p<0.001<br>q=0.002**
**Crypt loss**	**r=0,597<br>p=0.002<br>q=0.006**	r=-0,463<br>p=0.129<br>q=0.178	**r=-0,543<br>p=0.005<br>q=0.018**	r=0,144<br>p=0.482<br>q=0.530	r=0,287<br>p=0.164<br>q=0.501	r=-0,270<br>p=0.183<br>q=0.335	**r=-0,509<br>p=0.008<br>q=0.029**	r=-0,386<br>p=0.057<br>q=0.155	r=-0,267<br>p=0.187<br>q=0.412	**r=-0,414<br>p=0.035<br>q=0.097**	**r=0,638<br>p<0.001<br>q=0.002**	**r=1**

Supplementary Table S4 Correlations of S1P/Sphk2 with structural proteins and crypt architecture abnormalities. Pearson correlation coefficient (r), p-value (p), and False Discovery Rate (q). Each square represents the interaction between two variables, one horizontal and the other one vertical. Significant correlations (p<0.05 and q<0.1) are shaded and highlighted in bold
